# SLC27A5 inhibits cancer stem cells by inducing alternative polyadenylation of METTL14 in hepatocellular carcinoma

**DOI:** 10.1016/j.gendis.2024.101488

**Published:** 2024-12-10

**Authors:** Xin Tang, Junji Tao, Yuanyuan Liu, Deao Gong, Xuefeng Shan, Kai Wang, Ni Tang

**Affiliations:** a Department of Infectious Diseases, Key Laboratory of Molecular Biology for Infectious Diseases (Ministry of Education), Institute for Viral Hepatitis, The Second Affiliated Hospital, Chongqing Medical University, Chongqing 400016, China; b Department of Pharmacy, Bishan Hospital of Chongqing Medical University, Chongqing 402760, China

**Keywords:** Alternative polyadenylation, Hepatocellular carcinoma, METTL14, SLC27A5, Stemness

## Abstract

Solute carrier family 27 member 5 (SLC27A5/FATP5) is a liver-specific metabolic enzyme that plays a crucial role in fatty acid transport and bile acid metabolism. Deficiency of SLC27A5 promotes the progression of hepatocellular carcinoma (HCC) and is strongly associated with a poor prognosis. SLC27A5 exhibits noncanonical functions beyond its metabolic role; however, its specific mechanisms in hepatocarcinogenesis remain elusive and are therefore investigated in this study. Immunoprecipitation-mass spectrometry analysis showed that SLC27A5-interacting proteins were significantly enriched in alternative polyadenylation (APA). RNA-sequencing data provided evidence that SLC27A5 plays a global role in regulating APA events in HCC. Mechanistically, SLC27A5 facilitates the usage of the proximal polyadenylation site of *METTL14* by downregulating the expression of the APA-associated factor PABPC1, resulting in the shortening of the *METTL14*-3′UTR and the conversion of *METTL14*-UL to *METTL14*-US. In contrast to *METTL14*-UL, *METTL14*-US escapes the inhibitory effect of miRNA targeting, leading to increased METTL14 expression. *METTL14*-US upregulation by SLC27A5 suppressed the stemness of HCC. Therefore, low levels of SLC27A5 and METTL14 may serve as reliable biomarkers for identifying poor prognosis in patients with HCC. In conclusion, SLC27A5/PABPC1 inhibits HCC stemness via APA-regulated expression of METTL14, providing potential avenues for the development of novel therapeutic strategies.

## Introduction

Hepatocellular carcinoma (HCC) is a highly prevalent malignant tumor, with the third-highest mortality rate among all malignant tumors.^1^^,^^2^ The current lack of effective treatment options contributes to treatment failure, primarily due to the recurrence and metastasis of HCC.^3^^,^^4^ Liver cancer stem cells (LCSCs) within tumor tissues have been shown to drive HCC initiation, metastasis, and recurrence. Therefore, the elimination of LCSCs may offer a promising avenue for improving HCC treatment efficacy.^5^ However, the specific mechanisms by which LCSCs sustain stemness are incompletely understood. Gaining a comprehensive understanding of the molecular mechanisms involved in stemness maintenance in liver cancer cells may provide invaluable insights into HCC pathogenesis and lay a solid theoretical foundation for targeted therapy.

Alternative polyadenylation (APA) is a crucial post-transcriptional gene-regulatory mechanism. It regulates the utilization of proximal polyadenylation sites and distal polyadenylation sites, resulting in the generation of mRNA isoforms with different 3′-untranslated regions (3′-UTRs).^6^ These distinct isoforms exert diverse effects on mRNA stability, nuclear export, translation, localization, and protein localization of target genes.^7^, ^8^, ^9^, ^10^ Aberrant APA events have been widely observed in various types of cancer, including HCC, breast cancer, and pancreatic ductal adenocarcinoma.^11^, ^12^, ^13^, ^14^, ^15^ In these cancer cells, the altered 3′UTR length activates or suppresses oncogene expression^16^ and is strongly associated with stemness.^17^^,^^18^ Revealing the molecular mechanism underlying abnormal APA events in tumors is expected to provide new strategies to inhibit cancer cell stemness. Recent studies have shown that the dysregulation of APA factors significantly contributes to the occurrence of abnormal APA events and promotes HCC progression.^19^, ^20^, ^21^ Therefore, identifying dysregulated APA factors is essential to reveal the specific mechanisms of aberrant APA events in HCC.

As a nuclear-cytoplasmic shuttling protein, poly(A)-binding protein cytoplasmic 1 (PABPC1) plays an important role in regulating RNA metabolism, including mRNA translation,^22^ stability, nonsense-mediated decay,^23^ and hyperadenylation.^24^ PABPC1 is also critical in the regulation of 3′UTR-APA by promoting the use of distal PAS.^19^ PABPC1 directly regulates the APA of immunoglobulin mRNA and affects immunoglobulin secretion.^25^ Moreover, PABPC1 is highly expressed in various tumor tissues and is strongly associated with an unfavorable prognosis, particularly in HCC.^26^^,^^27^ Therefore, PABPC1 may be a potential target for the treatment of HCC. However, the mechanisms underlying aberrant PABPC1 expression and its regulatory role in differential APA events in HCC remain unclear.

Metabolic enzymes have the ability to regulate gene expression, which can, in turn, affect subsequent gene functions and cancer progression by protein-protein interactions, exerting kinase activity, or protein modifications.^28^, ^29^, ^30^, ^31^ Solute carrier family 27 member 5 (SLC27A5), also known as FATP5, is a fatty acid transport protein exclusively expressed in the liver and involved in bile acid metabolism.^32^ SLC27A5 deficiency promotes hepatic fibrosis and HCC progression via metabolic pathways.^33^, ^34^, ^35^ In addition, our group has discovered that SLC27A5 exerts a noncanonical function by interacting with IGF2BP3 to promote HCC metastasis via alternative splicing.^36^ However, it is unknown whether SLC27A5 regulates HCC progression via other noncanonical functions.

In this study, the analysis of SLC27A5-interacting proteins and the in-depth RNA-sequencing (RNA-seq) analysis demonstrated that SLC27A5 exerts global regulation of aberrant APA events in HCC. Notably, we found that SLC27A5 promotes the generation of a short 3'UTR isoform of methyltransferase-like protein 14 (*METTL14*) mRNA by interacting with PABPC1, which plays an anti-oncogenic role by upregulating METTL14 expression. Moreover, our results suggested that SLC27A5 contributes to the function of the shorter *METTL14* isoform in suppressing HCC stemness. Together, our data revealed a novel mechanism by which the SLC27A5-induced short *METTL14*-3′UTR isoform suppresses HCC stemness capacity, providing a potential therapeutic target for HCC.

## Materials and methods

### Cell culture

HepG2, SNU449, and PLC/PRF/5 cells were purchased from the American Type Culture Collection (ATCC; Manassas, VA, USA). MHCC-97H, HEK293, and HEK293T cells were obtained from the Cell Bank of the Chinese Academy of Sciences (Shanghai, China). SNU449 cells were cultured in Roswell Park Memorial Institute (RPMI) 1640 medium (Gibco, Grand Island, NY, USA) containing 10 % fetal bovine serum (Gibco, Rockville, MD, USA) and 1 % Penicillin-Streptomycin (MCE, New Jersey, USA), whereas all other cells were cultured in Dulbecco's modified Eagle's medium (DMEM, HyClone, Logan, UT, USA) containing 10 % fetal bovine serum and 1 % Penicillin-Streptomycin.

### Animal studies

All animal experiments were approved by the Medical Ethics Committee of Chongqing Medical University (approval No. 202309055) and conducted in accordance with guidelines issued by the committee. Eight-week-old male mice were randomly grouped (*n* = 7 per group). For the limiting dilution assay, different dilutions of single-tumor-cell suspensions (1 × 10^3^/1 × 10^4^/1 × 10^5^) were subcutaneously injected into the right flanks of the nude mice to establish a xenograft implantation model. The cells used for implantation were stably transfected with METTL14-UL or METTL14-US plasmids. At 50 days post-implantation, the mice were euthanized and the tumor tissues were excised for subsequent analysis.

*Slc27a5*^+/−^ mice on the C57BL/6 J background created using the CRISPR/Cas9 system (Cyagen Biosciences, KOCMP-26459-*Slc27a5-*B6N-VA*)* were crossed to generate *Slc27a5*^−/−^ mice. *Slc27a5*^−/−^ mice and wild type (WT) mice were treated with diethylnitrosamine (DEN) (75 mg/kg) and phenobarbital (PB) (at a concentration of 0.05 %) to induce HCC.^37^

### Clinical specimens

Forty-two paired HCC and adjacent normal tissue samples were obtained from patients treated at the Second Affiliated Hospital of Chongqing Medical University between 2019 and 2023. All patients provided informed consent, and the study was approved by the Institutional Ethical Review Board of Chongqing Medical University (approval No. 2023077). Patient-related information is provided in Table S1.

### Quantitative reverse transcription PCR (RT-qPCR)

RNA was extracted from hepatoma cells and clinical tissue specimens using TRIzol reagent (Invitrogen, Carlsbad, CA, USA) and reverse transcribed to cDNA using a PrimeScript™ RT Reagent Kit with gDNA Eraser (RR047A; TaKaRa, Tokyo, Japan). Target gene-specific primers were designed (Table S2), and the target genes were amplified using the SYBR Green qPCR Master Mix (1725121, Bio-Rad, California, USA). The amplification procedure was set according to the manufacturer's instructions. Target gene expression levels were determined using the 2^−ΔΔCt^ method.

### Calculation of distal polyadenylation site (dPAS) usage

Changes in dPAS usage were determined using a modified 2^−ΔΔCT^ method, as previously described.^38^ In brief, one pair of primers was designed to target the common part of METTL14 transcript isoforms (referred to as “common primers”) to amplify total transcripts, and one pair of primers was designed to target the distal part of the long isoform of METTL14 transcript (referred to as “distal primers”) to amplify long transcripts. The percentage of dPAS usage was determined by calculating ΔCt_total_ (ΔCt_distal_) = Ct_total_ (Ct_distal_) − Ct_β-actin,_ and ΔΔCt = ΔCt_distal_ − ΔCt_total_. Data are presented as fold changes normalized to the control, which were calculated as follows: ΔΔΔCt = ΔΔCt_average of target_  −  ΔΔCt_average of control_. An increase or decrease in dPAS usage was calculated as ± 2^−normalized ΔΔΔCt^. A negative value indicates mRNA with a shortened 3′UTR compared to the control.

### 3′ Rapid amplification of cDNA ends (3′ RACE) analysis

Total RNA was extracted from tissues or cells using TRIzol reagent (Invitrogen). The length of the *METTL14* mRNA 3′-end was assessed by 3′ RACE using a 3′ RACE kit (Sangon, Shanghai, China). Total cDNA was generated using a 3′-adaptor primer. The first PCR was performed using the *METTL14* F1 primer and 3′ RACE outer primer. Nested PCR was performed using the *METTL14* F2 primer and 3′ RACE inner primer. The PCR products were analyzed using 1 % agarose gel electrophoresis. The sequence of the gene-specific primers used for 3′ RACE is listed in Table S2.

### RNA immunoprecipitation (RIP) assay

RNA-binding proteins (RBPs) were immunoprecipitated using a Magnetic RIP kit (17–700; Millipore, USA), according to the manufacturer's instructions. Cells were lysed using RIP lysis buffer containing RNase inhibitor. An appropriate amount of protein A/G magnetic beads coated with anti-PABPC1 was added to the lysates, and the samples were incubated at 4 °C overnight for immunoprecipitation. The magnetic beads were washed with RIP wash buffer five times, followed by proteinase K treatment. RNA was extracted from the eluted immunoprecipitates using TRIzol. The regulatory relationships between PABPC1 and target RNAs were assessed using RT-qPCR. Table S2 lists the specific primers used for the RIP assay. Table S3 lists the antibodies used for the RIP assay.

### Western blotting

Proteins were extracted using cell lysis buffer (P0013; Beyotime Biotechnology, Shanghai, China), supplemented with 1 % protease inhibitor cocktail (#C0001; TargetMol, MA, USA), and denatured using sodium dodecyl sulfate polyacrylamide gel electrophoresis (SDS-PAGE) loading buffer (50 mM Tris, pH 6.8, 2 % SDS, 10 % glycerol, 1 % β-mercaptoethanol, 0.005 % bromophenol blue) at 95 °C for 10 min. The immunoprecipitates were separated by SDS-PAGE and transferred onto polyvinylidene difluoride membranes (Millipore). After being blocked with 5 % milk at room temperature for 1 h, the membranes were probed with primary antibodies at 4 °C overnight and then with the secondary antibody at room temperature for 1 h. Protein complexes were detected using Clarity™ Western ECL Substrate (Bio-Rad). Table S3 lists the antibodies used for western blotting.

### Co-immunoprecipitation (Co-IP)

Cells were transfected with plasmids for 48 h and then lysed with NP-40 lysis buffer (Beyotime) containing protease inhibitors (#C0001; TargetMol, MA, USA) for 30 min. After incubation with the indicated antibodies at 4 °C, the lysate was centrifuged to collect the supernatant. Protein A/G magnetic beads (Millipore) were added and protein-magnetic bead complexes were allowed to form for 3 h. The immunoprecipitates were washed, denatured, and immunoblotted with primary antibodies.

### *In vivo* ubiquitination assay

Cells were co-transfected with His-Ubiquitin (His-Ub) and the indicated plasmids. Before cells were harvested, MG132 (10 μM) was used to prevent protein degradation. After 48 h of transfection, cells were harvested using lysis buffer containing 1 % SDS, boiled for 30 min for denaturation, and subjected to ultrasonic cracking and centrifugation. The indicated antibodies were used to target proteins in the supernatants and incubated with protein A/G agarose beads for 4 h. The beads-captured complexes were washed four times and analyzed by western blotting. Protein ubiquitination levels were examined using anti-His antibodies.

### Glutathione S-transferase (GST) pull-down assay

GST-SLC27A5 and His-PABPC1 were purified using glutathione agarose beads (GE Healthcare, Piscataway, NJ, USA) and HisPur Ni-NTA magnetic beads (Thermo Fisher, MA, USA), respectively, and then co-incubated with glutathione agarose beads at 4 °C for 3 h to allow the formation of protein-magnetic bead complexes. The complexes were eluted with wash buffer (137 mM NaCl, 2.7 mM KCl, 10 mM Na_2_HPO_4_, 2 mM KH_2_PO_4_, and 0.5 % Triton X-100), boiled with 2 × SDS loading buffer, and analyzed using Coomassie staining and immunoblot analysis.

### Immunofluorescence staining (IF)

Cells were fixed in 4 % paraformaldehyde for 30 min and permeabilized with 0.5 % Triton X-100 at room temperature for 15 min. Subsequently, the cells were blocked with goat serum and incubated with the indicated antibodies at 4 °C for 16 h. Then, the cells were incubated with secondary fluorescence-coupled antibodies (ZSGB-BIO, Beijing, China) at room temperature for 2 h, and the nuclei were stained with 1 μg/mL 4′,6-diamidino-2-phenylindole (DAPI; Roche, Basel, Switzerland) for 5 min. The stained cells were scanned using a laser-scanning confocal microscope (Leica TCS SP8; Solms, Germany).

### Immunohistochemistry

Embedded liver tissue sections were hydrated in a graded ethanol series and antigenically repaired in Phosphate-Citrate Buffer. The sections were blocked in normal goat serum (ZSGB-BIO) for 1 h and incubated with primary antibodies at 4 °C overnight. After three washes with PBS, the sections were incubated with secondary goat-anti-rabbit or goat-anti-mouse IgG antibody (ZSGB-BIO) at room temperature for 1 h. Then, they were stained with 3,3′-diaminobenzidine (ZSGB-BIO) and scanned using a Pannoramic Scan 250 Flash or MIDI system. Images were acquired using Pannoramic Viewer 1.15.2 (3DHISTECH Kft., Budapest, Hungry).

### shRNA constructs

Small double-stranded hairpin RNAs (shRNAs) targeting PABPC1 were designed and ligated into the *Hpa*I and *Xho*I sites of the pLL3.7 lentiviral vector (kindly provided by Prof. Bing Sun, Center for Excellence in Molecular Cell Science, Chinese Academy of Sciences, Shanghai, China). pVSV-G, pCMV-Δ8.9, and recombined lentiviral vector pLL3.7 were co-transfected into HEK293T cells at a 2:3:4 ratio to package the lentiviruses using Lipo8000 (Beyotime). HepG2 or SNU449 cells were transduced with the packaged lentiviruses in the presence of polybrene (5 μg/mL). The oligos are listed in Table S2.

### Luciferase reporter assay

The wild type (WT) or mutated pGL3-METTL14-aUTRs and pRL-TK were co-transfected into cells using Lipofectamine 8000. After 24 h, the cells were treated with various microRNAs (ChemShine Biotechnology Inc, Shanghai, China). After 48 h, the cells were lysed and assayed for luciferase activity using a dual-luciferase assay (Promega, Madison, WI, USA). All assays were performed in triplicate.

### RNA fluorescence in situ hybridization (FISH) assay

A Cy3-labeled probe specific for *METTL14*-UL (5′-Cy3-CAACCGACAAAGCAGCCATA-3′) and a FAM-labeled miR-5009-3p probe (5′-FAM-TTTTGGACTTTCAGATTTAGGA-3′) were used for hybridization in HCC cells. The FISH assay was performed using an RNA Fluorescent In Situ Hybridization Kit (GenePharma, China), following the manufacturer's protocol. Images were acquired using a fluorescence microscope (Leica TCS SP8, Solms, Germany).

### Tumorsphere formation assay

PLC/PRF/5 and MHCC-97H cells were seeded into an ultra-low-attachment 6-well plate at a density of 5000 cells/well and cultured in HAM's F12 medium (Hyclone, Logan, UT, USA) supplemented with 20 ng/mL EGF (PeproTech, Cranbury, New Jersey, USA), 20 ng/mL FGF (PeproTech), and 1 × B27 (Gibco, NY, USA). After 14 days of culture, the tumor spheres were counted.

### Flow cytometry

Cells (1 × 10^7^) were suspended in prechilled PBS and then incubated with anti-CD90 and anti-CD44 for 30 min. Non-stained samples were used as negative controls. The cells were washed three times with prechilled PBS and analyzed using a FACSCanto II flow cytometer (BD Biosciences) and FlowJo software (Tree Star Inc.).

### RNA-seq analysis for APAlyzer

RNA-seq was performed at LC-BIO Biotech Ltd. (Hangzhou, China). Total RNA was extracted from parental or SLC27A5-knockout (KO) HepG2 cells (1 × 10^7^) using TRIzol reagent (Invitrogen). Sequencing libraries were prepared using the Illumina TruSeq Stranded Total RNA/Ribo-Zero Sample Prep Kit. Six samples were sequenced on an Illumina NovaSeq 6000. The RNA-seq data were deposited in the BioProject under accession number PRJNA935846 (https://dataview.ncbi.nlm.nih.gov/object/PRJNA935846). We used the algorithm APAlyzer (https://bioconductor.org/packages/release/bioc/html/APAlyzer.html) to characterize APA events using the RNA-seq data. APAlyzer uses the PolyA_DB database to identify poly(A) sites (PASs) and estimates the relative expression score of the first and last PASs in the 3′UTR of each gene for APA analysis. The difference in APA of a gene between parental and SLC27A5-KO RNA-seq is represented by the relative expression difference (RED). We used a *t*-test to assess the significance of differences in APA. Statistical significance was set at *P* < 0.05. Based on REDs and *P*-values, APAlyzer reports APA regulation as follows: “UP” indicates 3′UTR lengthening and “DN” indicates 3′UTR shortening. Volcano plots were used to visualize the results. Gene Ontology enrichment analysis was performed using the clusterProfiler package in R.

### CRISPR/Cas9-mediated knockout of SLC27A5

The single guide RNA sequence targeting SLC27A5 was cloned into a CRISPRv2 lentiviral vector expressing Cas9, which was provided by Prof. Ding Xue from Tsinghua University, Beijing, China. The lentivirus was then packaged and screened for stable knockout cell lines as described previously.^34^

### Online database analysis

Gene set enrichment analysis (GSEA) of The Cancer Genome Atlas (TCGA) HCC datasets was performed using GSEA 4.3.2 software. Correlations among gene expression levels in HCC were obtained from GSE25097. miRNAs binding to the *METTL14* 3′UTR were obtained from TargetScanHuman 8.0 (https://www.targetscan.org/vert_80/) and miRBD (https://mirdb.org/). Survival differences were validated at the gene expression level in TCGA HCC using Kaplan–Meier Plotter. Correlations among protein expression levels in HCC were obtained from PXD006512.

### Statistics and reproducibility

Data are expressed as mean ± SD. Statistical analyses were performed using GraphPad Prism7 (GraphPad Software Inc.). Student's *t*-test or a paired *t*-test was used for the comparison of two groups. One-way ANOVA followed by Tukey's test was used for all other variables. Correlations were analyzed using Pearson's correlation coefficient (R). Significance was set at *p* < 0.05.

## Results

### SLC27A5 directly binds to PABPC1 proteins and promotes its ubiquitinated degradation

**Figure 1:**
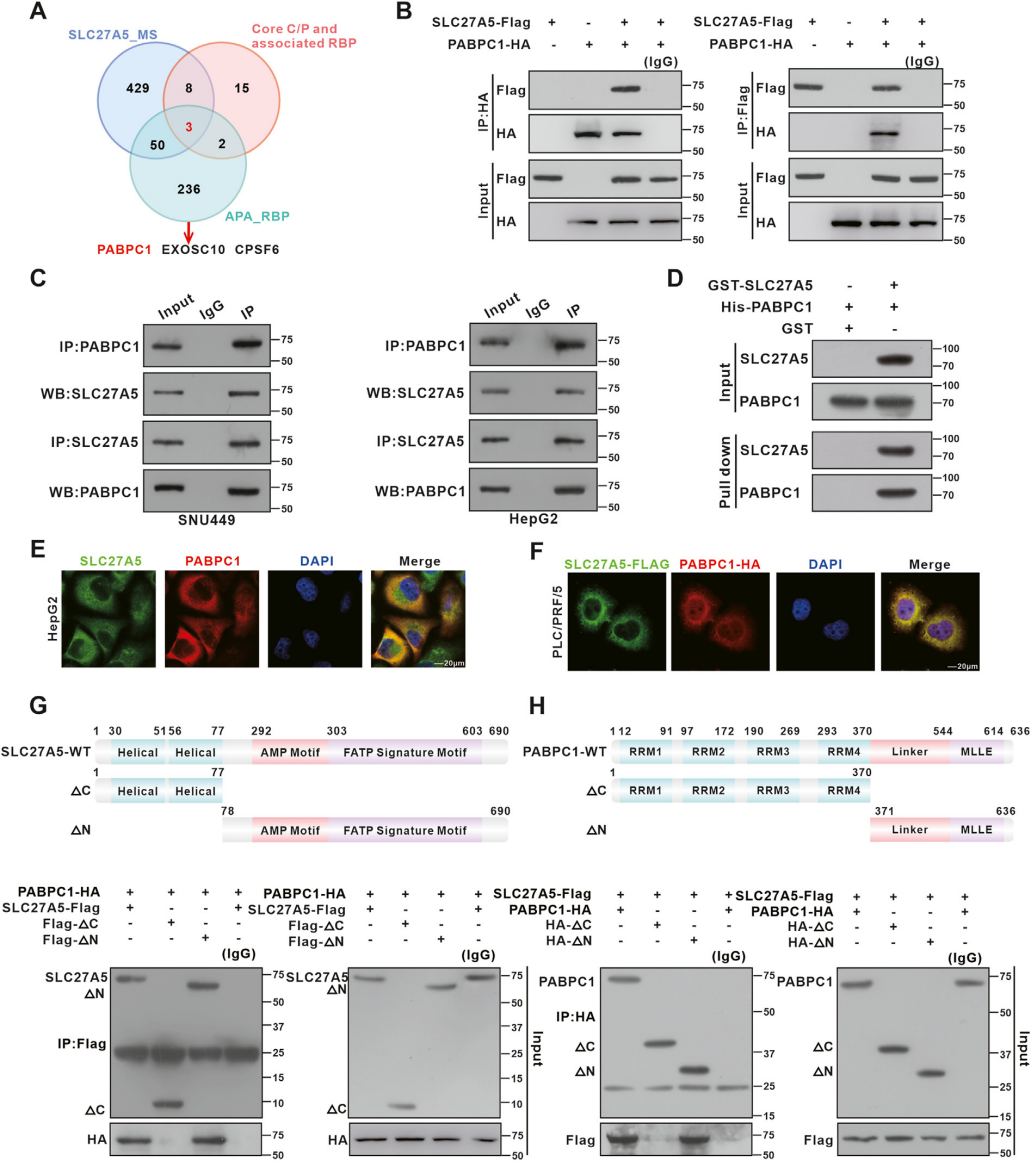
SLC27A5 directly binds to PABPC1 proteins and facilitates its degradation. **(A)** Proteins interacting with SLC27A5 were detected by IP-MS. APA-related RBPs (PMID: 22502866) and core cleavage/polyadenylation factors and associated RBP (PMID: 25906188) are illustrated in a Venn diagram. **(B)** Co-immunoprecipitation (Co-IP) of SLC27A5-Flag and PABPC1-HA using an anti-HA antibody (left) or an anti-Flag antibody (right) in HEK-293 cells. **(C)** Immunoblot analysis of Co-IP by anti-SLC27A5 and anti-PABPC1 in SNU449 cells (left) and HepG2 cells (right). **(D)** GST pull-down assays of the interaction between SLC27A5 and PABPC1. **(E)** Immunofluorescence staining to assess the subcellular co-localization of SLC27A5 and PABPC1 in HepG2 cells (scale bar: 20 μm). **(F)** Immunofluorescence staining was performed using an anti-Flag and anti-HA antibody in PLC/PRF/5 cells transfected with SLC27A5-Flag and PABPC1-HA to detect the subcellular co-localization of SLC27A5 and PABPC1 (scale bar: 20 μm). **(G, H)** Schematic representation of the SLC27A5 (G) and PABPC1 (H) deletion mutants, and Co-IP assays with the full-length PABPC1 and SLC27A5, respectively. The SLC27A5 deletion mutants covered amino acids 1–77 (ΔC) or 78–690 (ΔN). The PABPC1 deletion mutants covered amino acids 1–370 (ΔC) or 371–636 (ΔN).

In our previous study,^36^ we found that proteins interacting with SLC27A5 were mainly enriched in RNA-related biological processes, including alternative splicing and APA, suggesting a potential role of SLC27A5 in APA regulation. APA has been universally observed in cancers, but the underlying mechanism in HCC remains unclear.^39^ Therefore, we investigated the mechanism by which SLC27A5 governs APA in HCC. APA is regulated by pre-mRNA cleavage and polyadenylation (C/P) core factors and other RBPs. To identify APA-associated RBPs interacting with SLC27A5, we employed a combined screening process involving immunoprecipitation-mass spectrometry (IP-MS) of SLC27A5, APA core factors and related RBPs, and APA-associated RBPs. Initially, we identified PABPC1, EXOSC10, and CPSF6 as candidate SLC27A5-interacting proteins (Fig. 1A). Considering that PABPC1 exhibited the highest peptide count and coverage among the three proteins, we primarily focused on the interaction between PABPC1 and SLC27A5 (Fig. S1A). Co-IP experiments using HEK293 and HCC cells, which have high endogenous SLC27A5 levels, revealed the interaction between SLC27A5 and PABPC1 (Fig. 1B and C). GST pull-down assays confirmed the direct binding between these two proteins (Fig. 1D; Fig. S1B). Immunofluorescence assays revealed that SLC27A5 and PABPC1 colocalize mainly in the cytoplasm, with a lesser presence in the nucleus (Fig. 1E and F). To determine if the interaction between SLC27A5 and PABPC1 relied on its metabolic enzyme activity, we generated an enzyme-inactive form of SLC27A5, designated as S296A.^36^ Co-IP assays confirmed that the interaction was independent of its enzymatic activity (Fig. S1C). To further clarify the specific domains involved in the interaction, we next constructed truncated mutants based on the structures of SLC27A5 and PABPC1. The results indicated that there is an effective interaction between the C-terminal domain of SLC27A5 (aa 78–690) and the C-terminal region of PABPC1 (aa 371–636) (Fig. 1G and H).

Subsequently, we aimed to investigate the effect of SLC27A5 on PABPC1 expression, finding that PABPC1 mRNA levels remained unchanged (Fig. S2A), however, there was a significant decrease in PABPC1 protein expression after SLC27A5 overexpression (Fig. S2B–D). The primary protein degradation pathways in eukaryotic cells are the ubiquitin-proteasome and lysosomal pathways. Notably, we observed that the proteasome inhibitor MG132 reversed the down-regulation of PABPC1 induced by SLC27A5 overexpression, while the lysosomal inhibitor chloroquine (CQ) did not (Fig. S2E). These findings suggested that SLC27A5 may reduce PABPC1 expression via the ubiquitin-proteasome pathway.

Since E3-ubiquitin ligases are crucial for catalyzing ubiquitination by transferring ubiquitin proteins to the specific substrates, we focused on identifying the E3-ubiquitin ligase that mediates PABPC1 degradation through SLC27A5. A combined analysis of SLC27A5-interacting proteins and the UbiBrower database, which predicts E3-ubiquitin ligases for PABPC1, indicated that SLC27A5 may enhance PABPC1 ubiquitination through the E3-ubiquitin ligase RB Binding Protein 7 (RBBP7) (Fig. S3A). To test this hypothesis, we verified the interaction of SLC27A5, PABPC1, and RBBP7 by Co-IP experiments (Fig. S3B–D). SLC27A5 enhanced the interaction between PABPC1 and RBBP7 (Fig. S3C), leading to the RBBP7-mediated ubiquitination of PABPC1 (Fig. S3E). Together, the above results revealed that SLC27A5 facilitates the ubiquitinated degradation of PABPC1 through RBBP7, downregulating PABPC1 expression.

### SLC27A5 promotes the shortening of the *METTL14*-3′UTR by interacting with PABPC1

**Figure 2:**
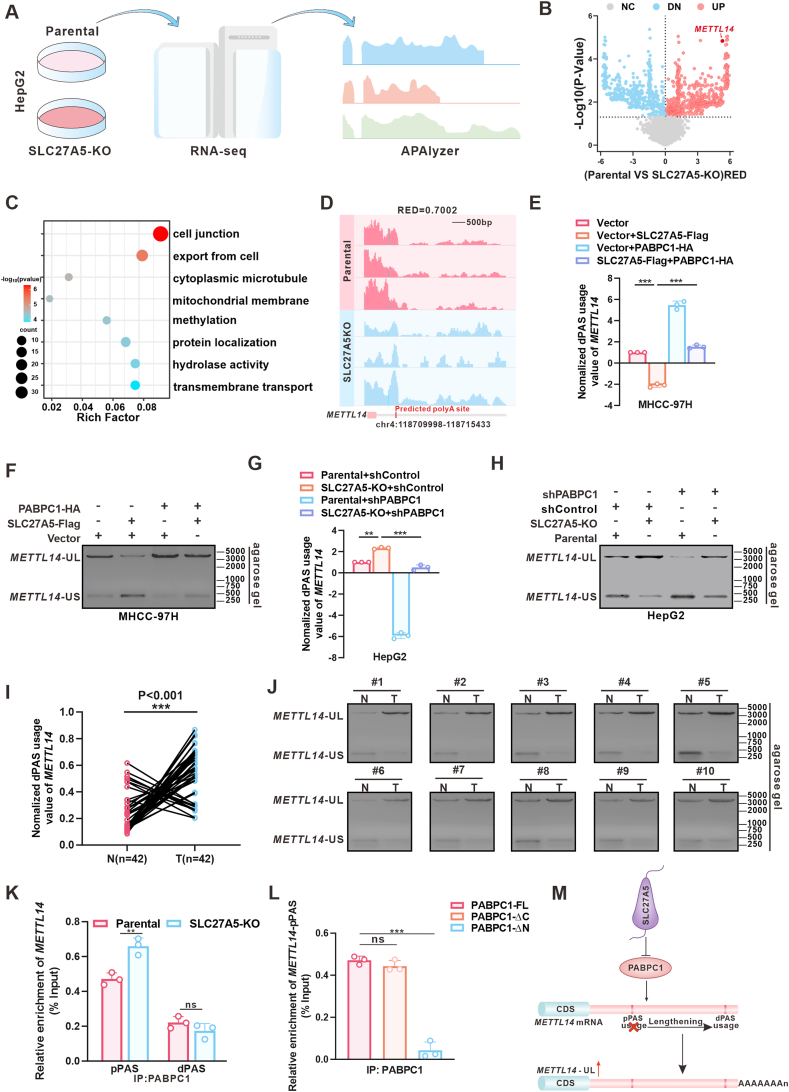
SLC27A5 promotes *METTL14*-3'UTR shortening by interacting with PABPC1. **(A)** Workflow of RNA-seq and APAlyzer analyses of parental or SLC27A5-KO HepG2 cells. **(B)** Volcano plot of APA events in parental and SLC27A5-KO HepG2 cells. Blue (DN) and red (UP) dots indicate the shortened and the lengthened transcripts, respectively. A Relative Expression difference (RED) represents the APA difference in a gene between groups. *p* ≤ 0.05. **(C)** GO enrichment analysis of genes with the altered 3'UTR lengths in SLC27A5-KO HepG2 cells. **(D)** Integrative Genomics Viewer (IGV) showed the shift of PASs in *METTL14* after SLC27A5 KO. The red box indicates proximal PAS predicted by DaPars. **(E**–**J)** qPCR analysis and 3′ RACE amplification showing the changes in dPAS usage of *METTL14* and *METTL14*-UL/S expression in SLC27A5-OE MHCC-97H cells transfected with PABPC1-HA or vector (E, F), SLC27A5-KO HepG2 cells transfected with PABPC1 shRNA or negative control shRNA (G, H), and tumor or corresponding non-tumorous tissues from patients with HCC (I, J). ∗∗*p* < 0.01; ∗∗∗*p* < 0.001. **(K)** PABPC1-RIP-qPCR showing the binding between proximal polyadenylation sites or distal polyadenylation sites of *METTL14* and PABPC1 in SLC27A5-KO HepG2 cells (*n* = 3). ∗∗*p* < 0.01. **(L)** RIP-qPCR showing the binding domain of *METTL14*-pPAS in PABPC1 (*n* = 3). ∗∗∗*p* < 0.001. **(M)** Model of SLC27A5 modulation of PABPC1 downregulation to inhibit S-to-L transition.

To investigate the differential APA events regulated by the SLC27A5/PABPC1 complex in HCC, we proceeded to gain insights into the significant 3'UTR alteration events regulated by SLC27A5 and then examine the dependency of these alterations on PABPC1. To accomplish this, we conducted an RNA-seq data analysis of SLC27A5-knockout and parental HepG2 cells using the bioinformatics algorithm APAlyzer^40^ (Fig. 2A). The findings revealed that 495 genes exhibited a significant shortening of their 3′UTR, referred to as distal-to-proximal shift, whereas 555 genes showed 3'UTR lengthening in response to SLC27A5 knockout (*p* < 0.05) (Fig. 2B). Subsequent functional annotation analysis revealed that these genes differentially regulated by SLC27A5 may have important roles in the biological process of cell junctions (Fig. 2C). Among the genes that were significantly enriched, *METTL14* was the most statistically significantly differentially expressed. Therefore, we focused on this gene and investigated whether *METTL14* mRNA undergoes differential APA events regulated by SLC27A5/PABPC1 in HCC. We generated *METTL14*-3′UTR profiles to confirm the lengthening of the *METTL14*-3′UTR due to SLC27A5 knockout (Fig. 2D), and designed primers (Table S2) to determine the expression of *METTL14* transcripts with the longer 3'UTR (*METTL14*-UL) and *METTL14* transcripts with the shorter 3'UTR (*METTL14*-US). Subsequently, RT-qPCR and nucleic acid gel analyses of 3′ RACE revealed a shift from *METTL14*-UL to *METTL14*-US in HCC cells overexpressing SLC27A5, attributed to a diminished utilization frequency of *METTL14*-dPAS. This shift could be reversed by PABPC1 overexpression (Figs. 2E and F; S4A, B). In contrast, reverse tendencies were observed in SLC27A5-KO cells and liver tumors of HCC patients (Figs. 2G–J; S4C, D). Moreover, the knockdown of PABPC1 partially decreased the usage of *METTL14*-dPAS and the expression of *METTL14*-UL in SLC27A5-KO cells, which verified that SLC27A5 shortened the length of the *METTL14*-3'UTR via down-regulating PABPC1 expression (Figs. 2G and H; S4C, D). RIP-qPCR analysis confirmed that SLC27A5 deficiency enhanced the recruitment of PABPC1 to the region around *METTL14*-pPAS, and this interaction was specifically mediated by the RNA-recognition motif (RRM) domain (1–370 aa) of PABPC1 (Fig. 2K and L). Finally, to investigate whether the function of SLC27A5 in regulating the APA of the *METTL14*-3'UTR depended on its enzymatic activity, we conducted 3′ RACE assay in HCC cells transfected with the SLC27A5-S296A mutant. Notably, the SLC27A5-S296A mutant, similar to the wild type (WT), was able to promote the conversion from the *METTL14*-UL to the *METTL14*-US (Fig. S4E). Collectively, these findings suggested that the SLC27A5 decreases the expression of PABPC1 and the utilization frequency of *METTL14*-dPAS, leading to the transformation of *METTL14*-UL to *METTL14*-US transcripts independent of its enzymatic activity (Fig. 2M).

### *METTL14*-US inhibits stemness in HCC both *in vitro* and *in vivo*

**Figure 3:**
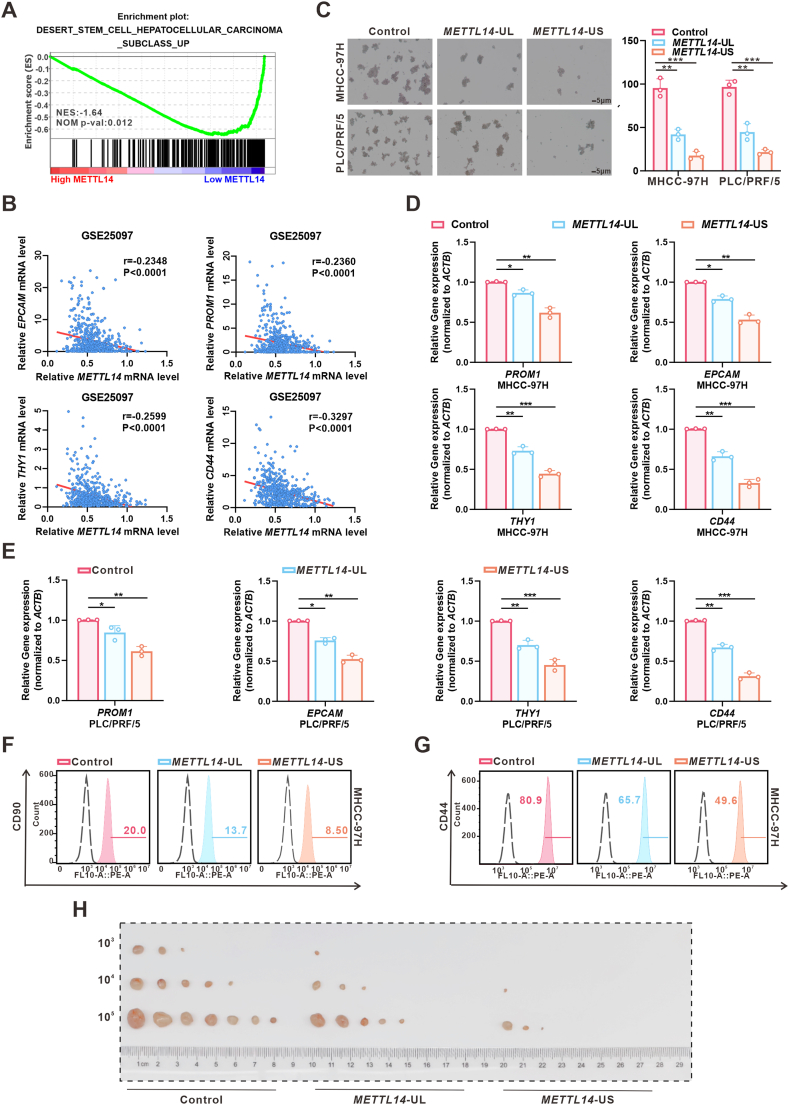
*METTL14*-US inhibits HCC stemness. **(A)** GSEA plot showing the correlation between low METTL14 expression and the regulation of HCC stemness. Black vertical lines indicate genes associated with METTL14. **(B)** Correlation analysis between *METTL14* and *EPCAM*, *PROM1*, *THY1*, or *CD44* based on the GEO database. **(C)** Representative images of sphere-forming assays in MHCC-97H and PLC/PRF/5 transfected with *METTL14*-UL/S. The quantification of the spheroids is shown in bar graphs. Scale bar, 5 μm (*n* = 3 independent replicates). ∗∗*p* < 0.01; ∗∗∗*p* < 0.001. **(D, E)** RT-qPCR analysis of *PROM1*, *EPCAM*, *THY1*, and *CD44* in MHCC-97H cells (D) and PLC/PRF/5 cells (E) transfected with *METTL14*-UL or *METTL14*-US (*n* = 3 technical replicates). ∗*p* < 0.05; ∗∗*p* < 0.01; ∗∗∗*p* < 0.001. **(F, G)** Representative images of flow-cytometric analysis of membrane CD90 (F) and CD44 (G) expression in the MHCC-97H cells transfected with vector, *METTL14*-UL or *METTL14*-US. **(H)** Limiting dilution assay of *METTL14*-UL/S-overexpressing MHCC-97H cells. Mice were subcutaneously injected with different cell numbers (1 × 10^5^, 1 × 10^4^, 1 × 10^3^). *n* = 7 per group.

To confirm whether *METTL14*-UL or *METTL14*-US has any biological functions in HCC, we investigated the potential biological roles of METTL14 in HCC development. First, our analysis of gene set enrichment analysis (GSEA) using The Cancer Genome Atlas (TCGA) Liver Hepatocellular Carcinoma (LIHC) data revealed a negative correlation between METTL14 and the regulation of liver cancer stemness (Fig. 3A). Second, correlation analysis using the Gene Expression Omnibus database also showed a negative correlation between *METTL14* mRNA levels and the expression of surface markers of HCC LCSCs, including *EPCAM*, *PROM1*, *THY1*, and *CD44* (Fig. 3B). These data suggested a potential role of different *METTL14* isoforms in the regulation of HCC stemness. Therefore, different *METTL14* isoforms were transfected into HCC cells (MHCC-97H and PLC/PRF/5). Sphere-forming assays showed that *METTL14*-US could markedly inhibit the tumor-forming capacity compared to *METTL14*-UL (Fig. 3C). In addition, we performed qRT-PCR to detect the impact of *METTL14*-UL/S on the levels of stemness-surface markers. The results showed that *METTL14*-US decreased the expression of *PROM1*, *EPCAM,* especially of *THY1* (*CD90*) and *CD44* (Fig. 3D and E). Therefore, we detected the percentage of the CD90^+^ or CD44^+^ cells in MHCC-97H cells overexpressing *METTL14*-US by flow cytometry analysis and found that the proportion of CD90^+^ or CD44^+^ cells was decreased (Fig. 3F and G). The above results showed that *METTL14*-US exhibited a more pronounced effect in curbing HCC stemness than *METTL14*-UL. These *in vitro* findings were further supported by limiting dilution assays and subcutaneous xenograft models, which demonstrated that *METTL14*-US significantly reduced the incidence of HCC cell xenografts in nude mice compared to *METTL14*-UL (Fig. 3H). Overall, these results clearly demonstrated that *METTL14*-US effectively inhibits the stemness of HCC cells both *in vitro* and *in vivo*.

### SLC27A5 regulates the ability of *METTL14*-US to inhibit stemness in HCC

**Figure 4:**
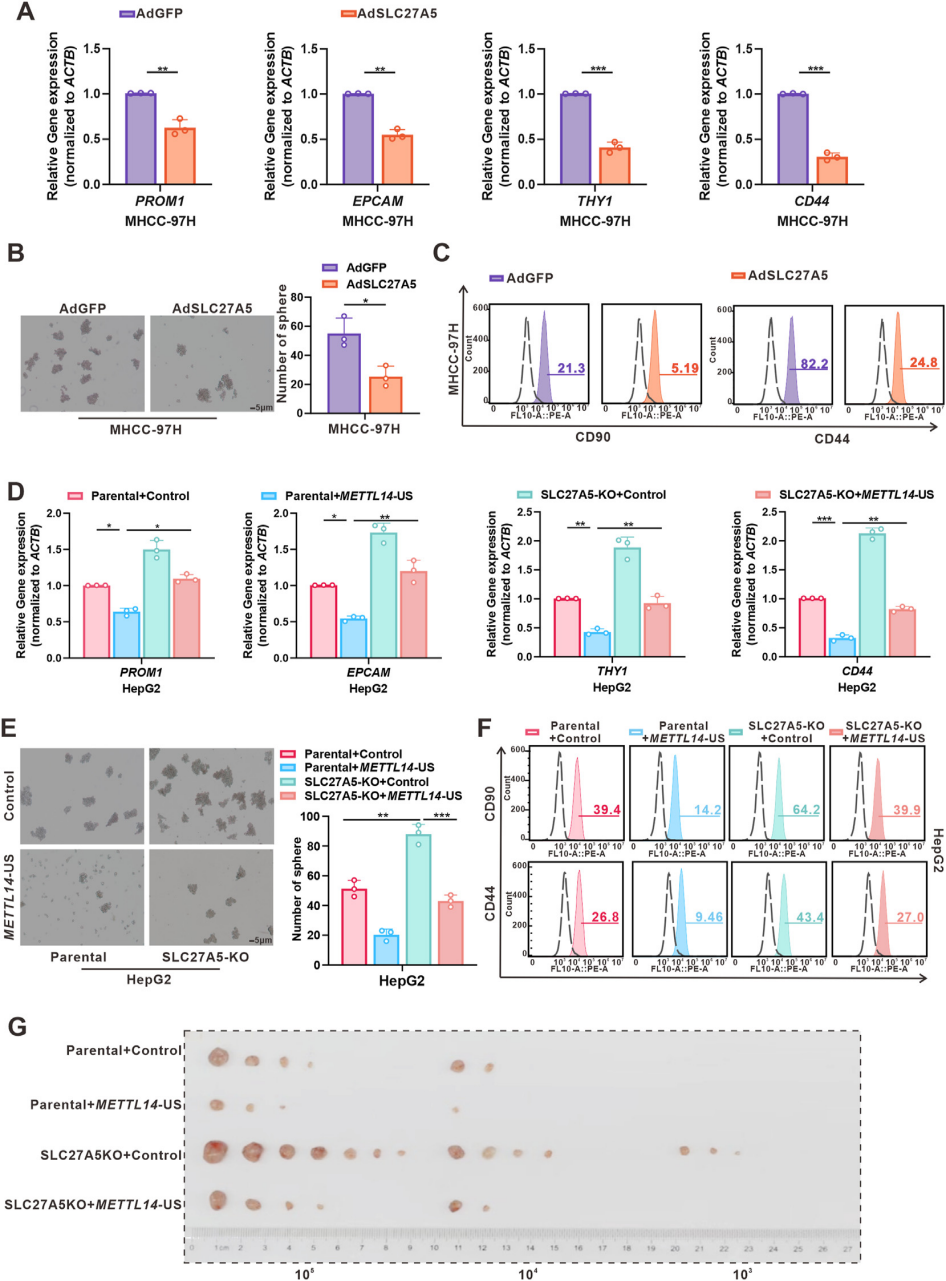
SLC27A5 inhibited HCC stemness by regulating *METTL14*-APA. **(A, B)** RT-PCR analysis of stemness-related indicators (A) and sphere-forming assays (B) in MHCC-97H cells infected with AdSLC27A5 (*n* = 3 independent replicates), ∗*p* < 0.05; ∗∗*p* < 0.01; ∗∗∗*p* < 0.001. **(C)** Flow-cytometric analysis of the percentage of CD90^+^ or CD44^+^ cells in MHCC-97H cells infected with AdSLC27A5. **(D)** SLC27A5-knockout HepG2 cells transfected with a control vector, or *METTL14*-US were used to detect stemness-related indicators by qPCR analysis (*n* = 3 technical replicates). ∗*p* < 0.05; ∗∗*p* < 0.01; ∗∗∗*p* < 0.001. **(E)** Representative images of sphere-forming assays in SLC27A5-KO HepG2 cells transfected with a control vector or *METTL14*-US. The quantification of spheroids is shown in bar graphs. Scale bar, 5 μm (*n* = 3 independent replicates). ∗∗*p* < 0.01; ∗∗∗*p* < 0.001. **(F)** Flow-cytometric analysis showing the percentage of CD90^+^ or CD44^+^ cells in SLC27A5-KO HepG2 cells transfected with a control vector or *METTL14*-US. **(G)** Limiting dilution assay of SLC27A5-KO HepG2 cells stably transfected with a control vector or *METTL14*-US. Mice were subcutaneously injected with different cell numbers (1 × 10^5^, 1 × 10^4^, 1 × 10^3^). *n* = 7 per group.

Since METTL14 is a downstream target of SLC27A5, we investigated whether SLC27A5 inhibited HCC stemness by regulating *METTL14*-APA. We first validated the impact of SLC27A5 on HCC stemness. The tumor-sphere formation rate and levels of stemness-surface markers were decreased in MHCC-97H and PLC/PRF/5 cells infected by AdSLC27A5 (Figs. 4A and B; S5A, B). The proportion of CD44^+^ or CD90^+^ cells was decreased in MHCC-97H cells overexpressing SLC27A5 (Fig. 4C). In parallel, the WT or S296A mutant SLC27A5 revealed negligible differences in their ability to suppress HCC stemness (Fig. S5C–E). The above findings suggested that SLC27A5 could inhibit HCC stemness independent of its enzyme activity. Meanwhile, we also observed that SLC27A5 depletion markedly enhanced the tumor-forming capacity in HepG2 and SNU449 cells, which was rescued by the re-expression of *METTL14*-US (Figs. 4D–F; S5F–H). This indicated that the impact of *METTL14*-US on HCC stemness was regulated by SLC27A5. To further investigate *in vivo*, we performed a limiting dilution assay to assess the tumor-initiating ability of SLC27A5-KO HepG2 cells stably transfected with *METTL14*-US. Depletion of SLC27A5 significantly enhanced HCC stemness, which was diminished upon re-expression of *METTL14*-US (Fig. 4G). Taken together, these results suggested that SLC27A5 inhibits HCC stemness by upregulating *METTL14*-US expression levels, independent of its enzyme activity.

### *METTL14*-US escapes from miRNA-mediated repression and leads to enhanced METTL14 expression

**Figure 5:**
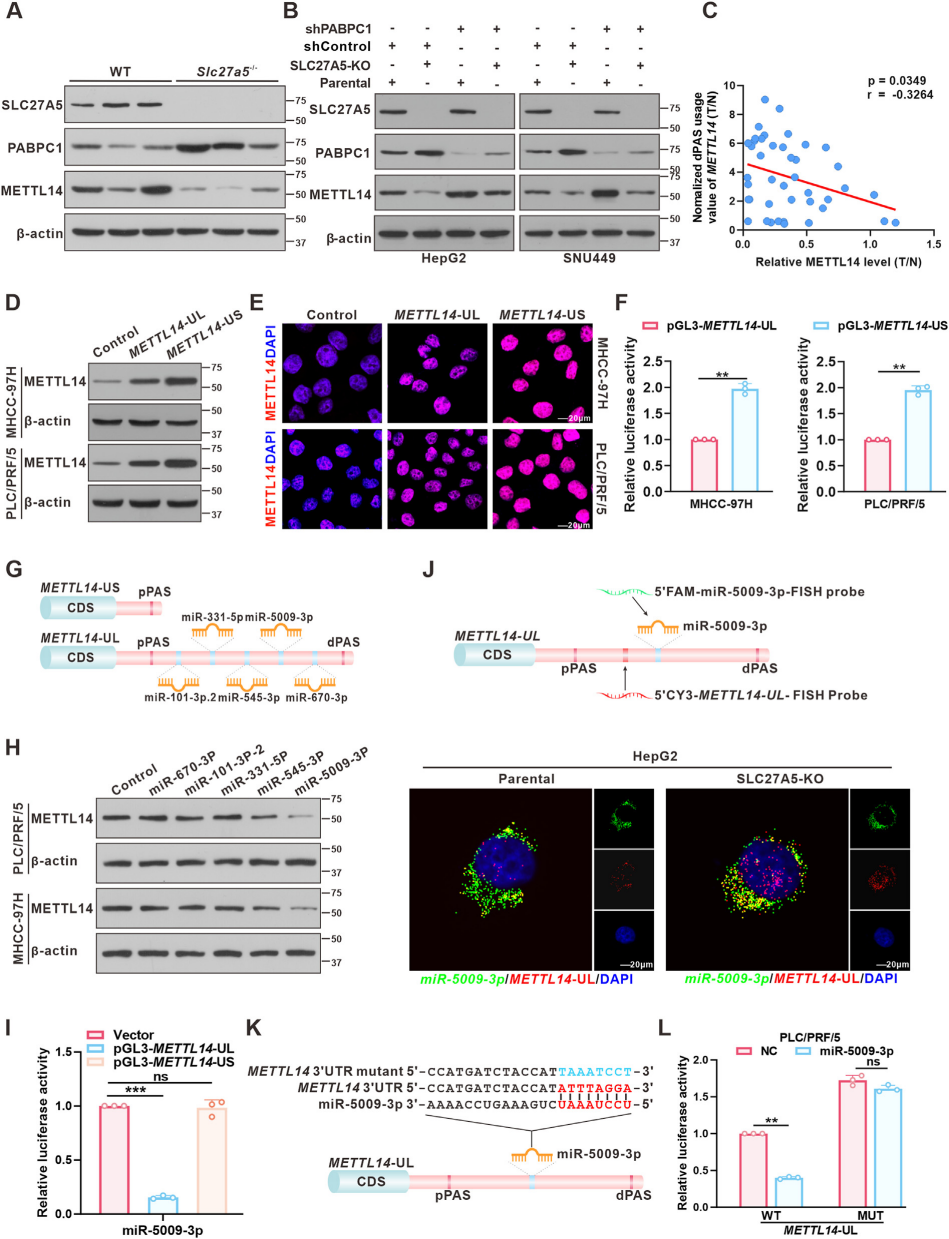
*METTL14*-US escapes from miRNA-mediated repression and promotes METTL14 protein expression. **(A)** Immunoblots of SLC27A5, PABPC1, and METTL14 in liver tumors of DEN/CCL4-induced WT and *Slc27a5*^−/−^ mice (*n* = 3 per group). **(B)** Western blot analysis of SLC27A5, PABPC1, and METTL14 in SLC27A5-KO cells transfected with PABPC1 shRNA or negative control shRNA. **(C)** The correlation between normalized dPAS usage and normalized METTL14 protein levels in patients with HCC (*n* = 42, Pearson correlation). ∗*p* < 0.05. **(D, E)** Immunoblots (D) and IF staining (E) of METTL14 in PLC/PRF/5 cells and MHCC-97H cells transfected with *METTL14-*UL or *METTL14*-US. **(F)** Luciferase activity of reporters carrying *METTL14*-UL/S in PLC/PRF/5 and MHCC-97H cells (*n* = 3 independent replicates). ∗∗*p* < 0.01. **(G)** Schematic illustration of *METTL14*-UL and *METTL14*-US. The blue vertical lines represent predicted miRNA-binding sites. **(H)** Western blot analysis of METTL14 in PLC/PRF/5 and MHCC-97H cells transfected with predicted miRNAs. **(I)** Luciferase reporter assay of reporters carrying *METTL14*-UL/S in PLC/PRF/5 cells co-transfected with miR-5009-3p (*n* = 3 independent replicates). ∗∗∗*p* < 0.001. **(J)** RNA FISH of *METTL14*-UL (red) and miR-5009-3p (green) in parental/SLC27A5-KO cells showing that *METTL14* and miR-5009-3p are co-localized in the cytoplasm. Nuclei are stained with DAPI (scale bar: 20 μm). **(K)** The predicted binding site for miR-5009-3p on *METTL14*-UL and the sequence of the mutant were established. **(L)** Luciferase activity of *METTL14*-UL-WT and *METTL14*-UL-MUT in PLC/PRF/5 cells co-transfected with miR-5009-3p (*n* = 3 independent replicates). ∗∗*p* < 0.01.

Next, we aimed to uncover the mechanisms underlying the varying effects of *METTL14*-UL/S, established via APA, on the regulation of HCC stemness. Abnormal 3'UTR-APA events primarily modulate the protein expression levels of target genes in the majority of tumors.^6^^,^^13^ Therefore, we hypothesized that *METTL14* pre-mRNA APA regulated by SLC27A5/PABPC1 may influence METTL14 expression. To verify this, we first conducted Western blot analyses of *Slc27a5*^−/−^ mice and of SLC27A5-KO cells treated with shRNA targeting PABPC1. The results showed that the absence of SLC27A5 decreased METTL14 expression levels (Fig. 5A and B), which could be reversed by PABPC1 knockdown (Fig. 5B). Additionally, METTL14 expression levels were closely associated with the transition between *METTL14*-UL and *METTL14*-US (Fig. 5C). Moreover, immunoblotting and immunofluorescence assays revealed that *METTL14*-US significantly enhanced METTL14 expression compared to *METTL14*-UL (Fig. 5D and E). These data indicated that the regulation of METTL14 expression by SLC27A5/PABPC1 is mediated via the conversion of *METTL14*-UL/S.

To further investigate the mechanism of *METTL14*-UL and *METTL14*-US in regulating METTL14 expression, we performed a luciferase activity assay by cloning the 3'UTR of the *METTL14* isoforms into a luciferase reporter vector. The results showed more vigorous luciferase activity in the reporter plasmid containing *METTL14*-US (Fig. 5F). Further analysis using TargetScan revealed that *METTL14*-UL contains multiple potential miRNA-binding sites compared to *METTL14*-US (Fig. 5G). Next, the predicted miRNAs were transformed into PLC/PRF/5 and MHCC-97H cells; we found that miR-5009-3p significantly downregulated METTL14 protein expression (Fig. 5H). A luciferase activity assay showed that miR-5009-3p inhibited the luciferase activity of *METTL14*-UL, but had no significant effect on *METTL14*-US (Fig. 5I). RNA FISH assays revealed a significant increase in the co-localization of *METTL14*-UL mRNA with miR-5009-3p in the cytoplasm following SLC27A5 depletion (Fig. 5J). Subsequently, we mutated the site where miR-5009-3p bound to *METTL14*-UL (Fig. 5K) and performed a luciferase activity assay. The result showed that mutant plasmids produced higher luciferase activity than plasmids carrying the wild type (WT), which confirmed the binding of *METTL14*-UL and miR-5009-3p (Fig. 5L). Together, these data indicated that *METTL14*-US mRNA is stabilized by evading miRNA-mediated silencing, which results in the upregulation of METTL14.

### Correlations among SLC27A5, PABPC1, and METTL14 expression in HCC specimens

**Figure 6:**
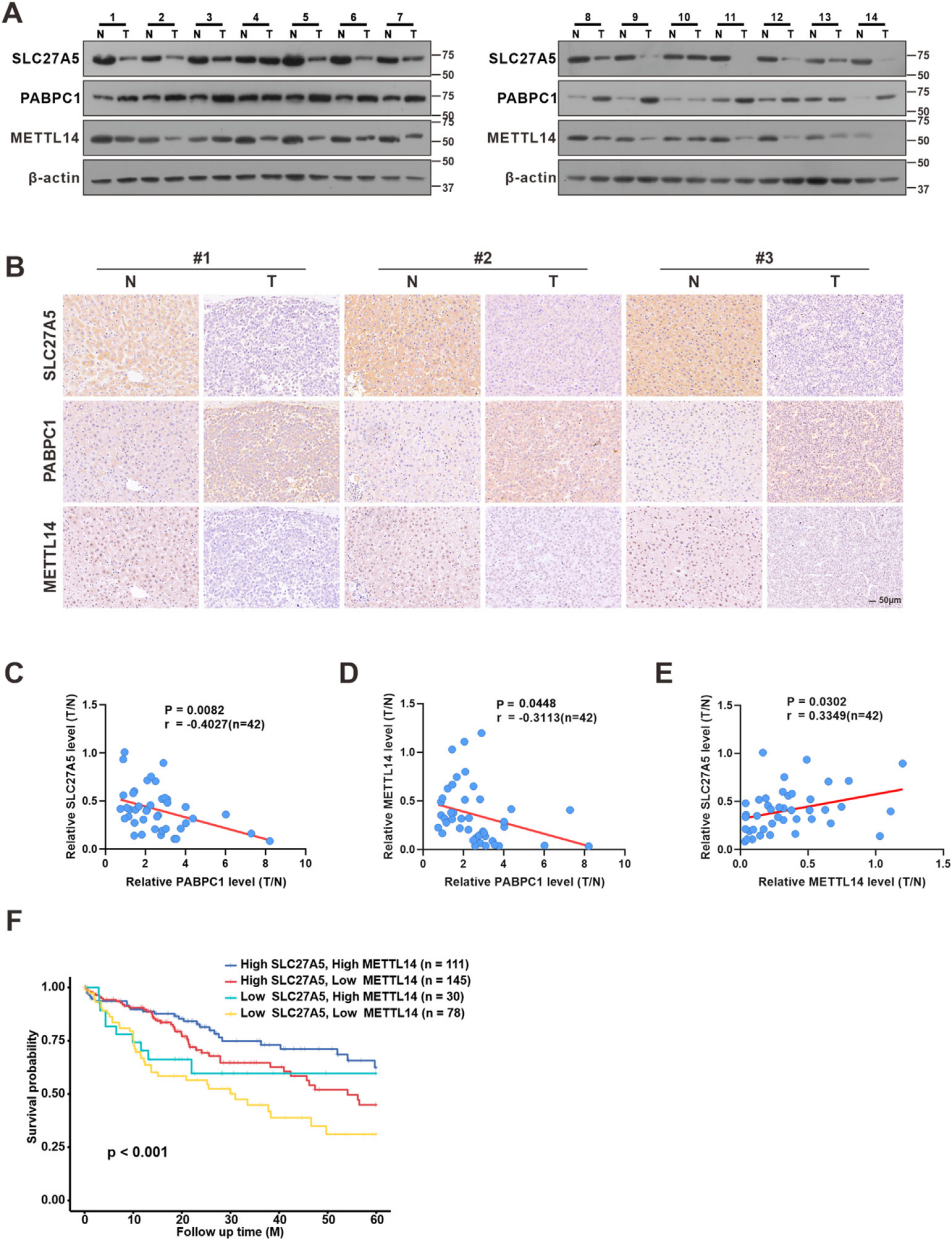
SLC27A5, PABPC1, and METTL14 expression in HCC specimens. **(A, B)** Expression of SLC27A5, PABPC1, and METTL14 in human liver tumor tissues and corresponding non-tumor tissues was detected by western blotting in 14 HCC patients (A) and by immunohistochemistry in consecutive sections of three pairs of HCC liver tissues (B). **(C**–**E)** Correlation analysis of indicated proteins in tumor and surrounding tissues from 42 HCC patients (also seen in figure S6A). The correlation analysis between SLC27A5 and PABPC1 (C), PABPC1 and METTL14 (D), SLC27A5 and METTL14 (E). **(F)** The Kaplan–Meier Plotter database was used to analyze the overall survival rate of patients (*n* = 364) stratified by SLC27A5 and METTL14 expression.

We further examined the expression levels of SLC27A5, PABPC1, and METTL14 in HCC and corresponding adjacent normal tissues. Western blot (*n* = 42) and immunohistochemistry (*n* = 10) analyses indicated a significant downregulation of SLC27A5 and METTL14 expression in HCC tissues compared to normal tissues. PABPC1 expression exhibited an opposite trend, with higher expression levels observed in HCC tissues than in normal tissues (Figs. 6A and B; S6A, B). Correlation analysis demonstrated a positive correlation between SLC27A5 and METTL14 (r = 0.3349, *p* = 0.0302), whereas PABPC1 showed a negative correlation with both SLC27A5 (r = −0.4027, *p* = 0.0082) and METTL14 (r = −0.3113, *p* = 0.0448) (Fig. 6C–E), which was consistent with the results of correlation analysis using the PRoteomics IDEntifications (PRIDE) database (Fig. S6C–E). Moreover, survival analysis showed that patients with lower levels of SLC27A5 and METTL14 had poorer overall survival (Fig. 6F). Collectively, these data supported that SLC27A5 deficiency facilitates the expression of PABPC1 and short 3'UTR isoforms of *METTL14*, thereby inhibiting METTL14 expression and promoting tumor progression.

## Discussion

Metabolic enzymes play a crucial role in tumor progression via metabolic reprogramming. Accumulating studies have explored the multiple regulatory mechanisms of metabolic enzymes in cancer cells, which can affect tumor progression through noncanonical functions without affecting normal metabolism. For instance, glycolytic enzymes Phosphofructokinase 1 and Enolase 1 have been found to directly bind to transcription factors and induce the expression of target genes, whereas Aldolase A and Lactate Dehydrogenase A take part in various epigenetic processes through protein interactions.^41^, ^42^, ^43^, ^44^ In addition, Pyruvate Kinase M2 and phosphoenolpyruvate carboxykinase 1 can act as kinases and contribute to tumor progression by phosphorylating and modifying substrate proteins.^45^^,^^46^ Despite the crucial roles of metabolic enzymes in gene expression, there has been limited exploration of their non-classical functions at the post-transcriptional level.

**Figure 7:**
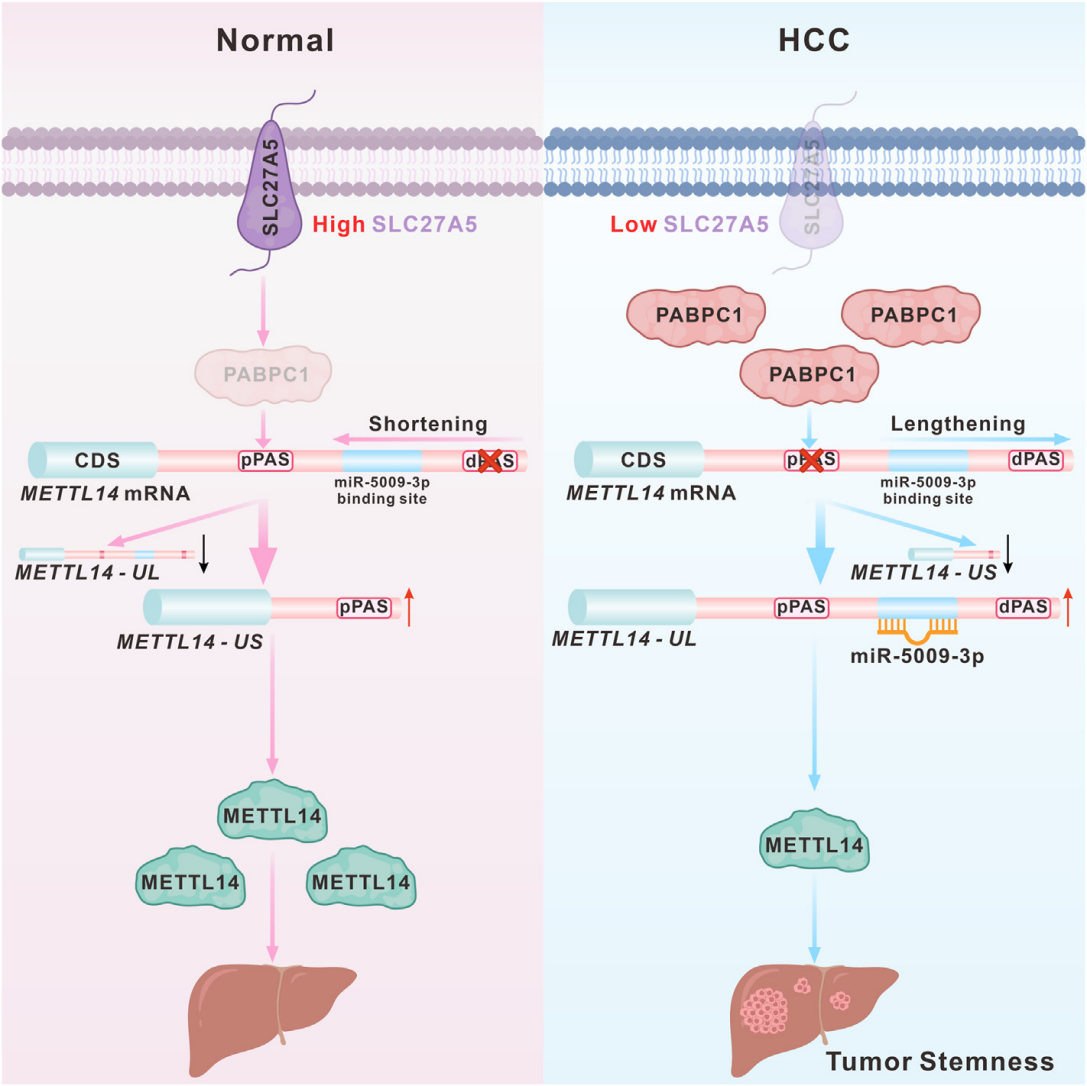
The mechanistic model of the study. Normally, SLC27A5 interacts with PABPC1, partially downregulating PABPC1 expression. In HCC, low SLC27A5 expression inhibits METTL14 expression by PABPC1-mediated APA of *METTL14* and enhances HCC stemness.

In this study, we found that SLC27A5 regulates the APA of the *METTL14*-3'UTR by interacting with PABPC1, leading to the generation of short 3'UTR isoforms of *METTL14*, which evades miRNA-mediated inhibition and consequently enhances METTL14 abundance, ultimately inhibiting HCC stemness (Fig. 7). These findings reveal a previously unknown noncanonical function of SLC27A5, an enzyme involved in lipid metabolism, in the regulation of APA at the post-transcriptional level. This study expands our understanding of the noncanonical functions of metabolic enzymes and deepens the intrinsic link between metabolic enzymes and the post-transcriptional processing of genes.

Several metabolic enzymes exert noncanonical functions, regulating the ubiquitin-dependent degradation and stability of interacting proteins. For example, fructose-1, 6-bisphosphatase 1 enhances Notch1 ubiquitination, promoting its proteasomal degradation via the FBXW7 pathway.^47^ Similarly, fructokinase C blocks p62 ubiquitination and its aggregation with Keap1, inhibiting NRF2 activation-dependent antioxidative stress gene expression, cell survival, and HCC development.^48^ Here, we found that SLC27A5 inhibited HCC stemness by interacting with PABPC1, independent of its enzymatic activity, revealing a critical noncanonical role of SLC27A5 in regulating HCC stemness. Notably, SLC27A5 interacted with PABPC1 and downregulated PABPC1 expression without affecting PABPC1 mRNA levels. Mechanistically, SLC27A5 bound to PABPC1, thereby enhancing the interaction between PABPC1 and RBBP7, which decreased the protein stability of PABPC1 through the ubiquitin-proteasome pathway. These findings uncover a novel mechanism for the downregulation of PABPC1 expression in HCC. However, further research is needed to identify additional ubiquitinating or deubiquitinating enzymes involved in SLC27A5-regulated PABPC1 degradation.

APA is primarily regulated by RBPs.^18^^,^^49^^,^^50^ Mechanistically, the aberrant expression of RBPs often leads to changes in APA, thereby promoting oncogenic transformation.^51^ We uncovered that PABPC1 expression is upregulated in HCC and mediates the noncanonical function of SLC27A5 in regulating the APA of the *METTL14*-3'UTR. PABPC1 plays an essential role in mRNA processing by interacting with multiple proteins and mRNAs.^52^ PABPC1 has been reported to play a vital role in regulating the APA process, and its degree of regulation is comparable to that of PABPN1.^19^ Moreover, cross-linking IP-seq analysis has shown that PABPC1 can directly bind to the PAS site in the 3'UTR region of numerous mRNAs.^53^ Several studies have suggested that hnRNPLL exerts the function of APA in plasma cells by interacting with PABPC1, which directly binds to immunoglobulin mRNA to promote the conversion of mIgH to sIgH.^25^ However, the specific role of PABPC1 in APA and its target genes in HCC remained unclear. We identified PABPC1 as an APA factor. It inhibited the shortening of the *METTL14*-3'UTR upon SLC27A5 depletion, suggesting the noncanonical function of SLC27A5. The mechanism of other PABPC1-regulated genes contributing to the moonlighting function of SLC27A5 is worth further investigation.

METTL14 functions as a tumor suppressor in HCC and is strongly associated with the patient prognosis.^54^ As a member of the methyltransferase complex, METTL14 is involved in N6-methyladenosine (m6A) modifications via its interaction with METTL3. We analyzed SLC27A5/PABPC1-regulated APA events in HCC cells using RNA-seq data and focused on *METTL14*-3'UTR APA. We identified a novel short 3'UTR isoform of *METTL14*(*METTL14*-US) in cells and tissues with high SLC27A5 levels. Furthermore, this newly identified transcript upregulated METTL14 expression and inhibited HCC stemness *in vitro* and *in vivo*. These results revealed that APA-induced *METTL14*-US is important for abnormal METTL14 expression and acts as a crucial anti-oncogenic driver in HCC progression. Moreover, overexpression of *METTL14*-US remarkably inhibited the HCC stemness after SLC27A5 depletion, suggesting that the inhibitory function of SLC27A5 in HCC stemness is partly mediated by *METTL14*-US. It also suggests that inhibiting the prolongation of the *METTL14*-3'UTR or promoting the upregulation of *METTL14*-3US may be a promising therapeutic strategy. Indeed, antisense oligonucleotides can specifically bind to and mask the distal PAS signals at specific distal PAS sites in precursor mRNAs, allowing the proximal PAS sites to be preferentially cleaved and tailed. This generates more stable mRNAs and upregulates protein expression.^55^ Antisense oligonucleotides restored the mRNA and protein levels of STMN2 in a dose-dependent manner by targeting the splicing and polyadenylation sites of STMN2, ultimately improving neuronal axonal regeneration.^56^ Therefore, targeting the poly(A) signal of METTL14 could open up new avenues for the development of therapeutic strategies.

In summary, this study revealed a previously unknown moonlighting function of SLC27A5 in regulating APA to suppress HCC stemness, highlighting a potential link between metabolic enzymes and mRNA post-transcriptional processing in cancer. SLC27A5 interacts with PABPC1 and hinders the occupation of *METTL14*-pPAS by PABPC1, which promotes the production of the transcriptional isoform *METTL14*-US and enhances METTL14 protein abundance. The discovery of the SLC27A5-PABPC1-METTL14 axis deepens our understanding of the metabolic network of cancer and mRNA alternative polyadenylation. Targeting SLC27A5-induced APA or *METTL14*-US may be a novel therapeutic approach for impeding HCC progression.

## CRediT authorship contribution statement

**Xin Tang:** Conceptualization, Data curation, Validation, Writing – original draft, Writing – review & editing. **Junji Tao:** Data curation, Software, Validation. **Yuanyuan Liu:** Data curation, Software. **Deao Gong:** Data curation, Software. **Xuefeng Shan:** Conceptualization, Supervision. **Kai Wang:** Supervision, Validation, Writing – original draft, Writing – review & editing. **Ni Tang:** Funding acquisition, Project administration, Supervision, Validation, Writing – original draft, Writing – review & editing.

## Data availability

The data that support the findings of this study are available from the corresponding author upon reasonable requests.

## Funding

This work was supported by the National Key Research and Development Program of China (No. 2023YFC2306800); The National Natural Science Foundation of China (No. 82272975, 82073251, 82304288); Natural Science Foundation of Chongqing (No. CSTB2024NSCQ-KJFZMSX0016); the Innovative and Entrepreneurial Team of Chongqing Talents Plan; Chongqing Medical Scientific Research Project, China (Joint Project of Chongqing Health Commission and Science and Technology Bureau, No. 2023DBXM007); the Future Medical Youth Innovation Team of Chongqing Medical University (China) (No. W0036, W0101); Senior Medical Talents Program of Chongqing for Young and Middle-aged (China); the Kuanren Talents Program and Joint Project of Pinnacle Disciplinary Group of the Second Affiliated Hospital of Chongqing Medical University (China).

## Conflict of interests

The authors declare no potential conflict of interests.
