# Supplementary material for: SLC27A5 inhibits cancer stem cells by inducing alternative polyadenylation of METTL14 in hepatocellular carcinoma
Source: Genes Dis. 2024 Dec 10;12(4):101488. doi: 10.1016/j.gendis.2024.101488 (PMC12033915; doi:10.1016/j.gendis.2024.101488)
Supplement: Multimedia component 1 [file mmc1.docx]

**Supplementary Material**

**SLC27A5 inhibits cancer stem cells by inducing alternative polyadenylation of METTL14 in hepatocellular carcinoma**

Xin Tang^a,1^, Junji Tao^a,1^, Yuanyuan Liu^a^, Deao Gong^a^, Xuefeng Shan^b,^**^*^**, Kai Wang^a,^**^*^**, Ni Tang^a,^**^*^**

**Supplementary figures, figure legends, and tables**

Figure S1. SLC27A5 interacts with PABPC1 independently of its enzymatic activity.

Figure S2. SLC27A5 overexpression downregulates PABPC1 expression. Figure S3. SLC27A5 promotes the ubiquitination of PABPC1 via RBBP7.

Figure S4. SLC27A5 promotes *METTL14*-3'UTR shortening via PABPC1.

Figure S5. SLC27A5 inhibits HCC stemness via *METTL14*-US, not depending on its enzymatic activity.

Figure S6. Correlations among SLC27A5, PABPC1, and METTL14 expression in HCC specimens.

Table S1. Clinical characteristics of HCC patients.

Table S2. Primer sequences are used in this study.

Table S3. Antibodies and plasmids are used in this study.

**Supplementary Information**

**
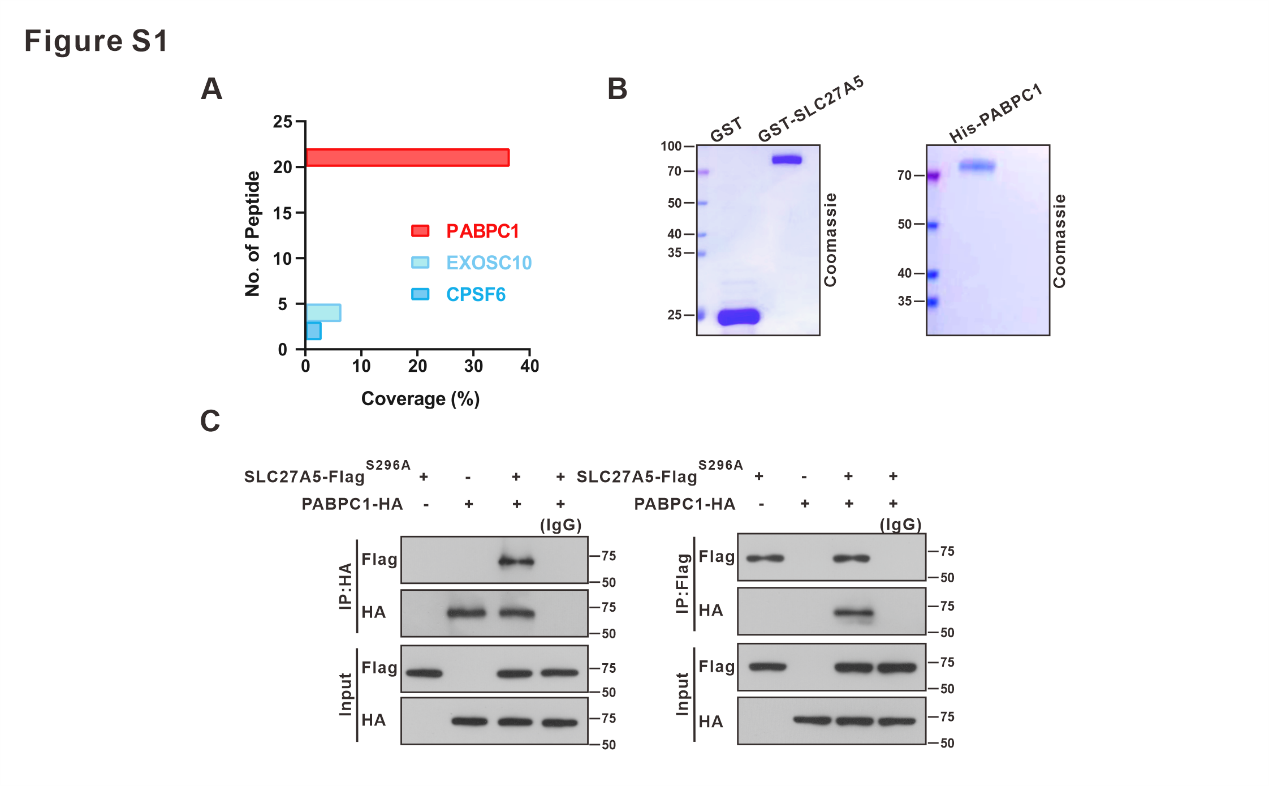
**

**Figure S1** SLC27A5 interacts with PABPC1 independently of its enzymatic activity. **(A)** APA-associated RBPs identified among potential SLC27A5-interacting proteins are illustrated with coverage (%) and peptide count. **(B)** The left panel shows the purified SLC27A5-GST protein and the right panel shows the purified PABPC1-His protein (Coomassie blue staining). **(C)** Co-IP assay of SLC27A5-Flag (S296A) and PABPC1-HA performed with anti-HA (left) or anti-Flag (right) in HEK-293 cells. lgG served as a control.


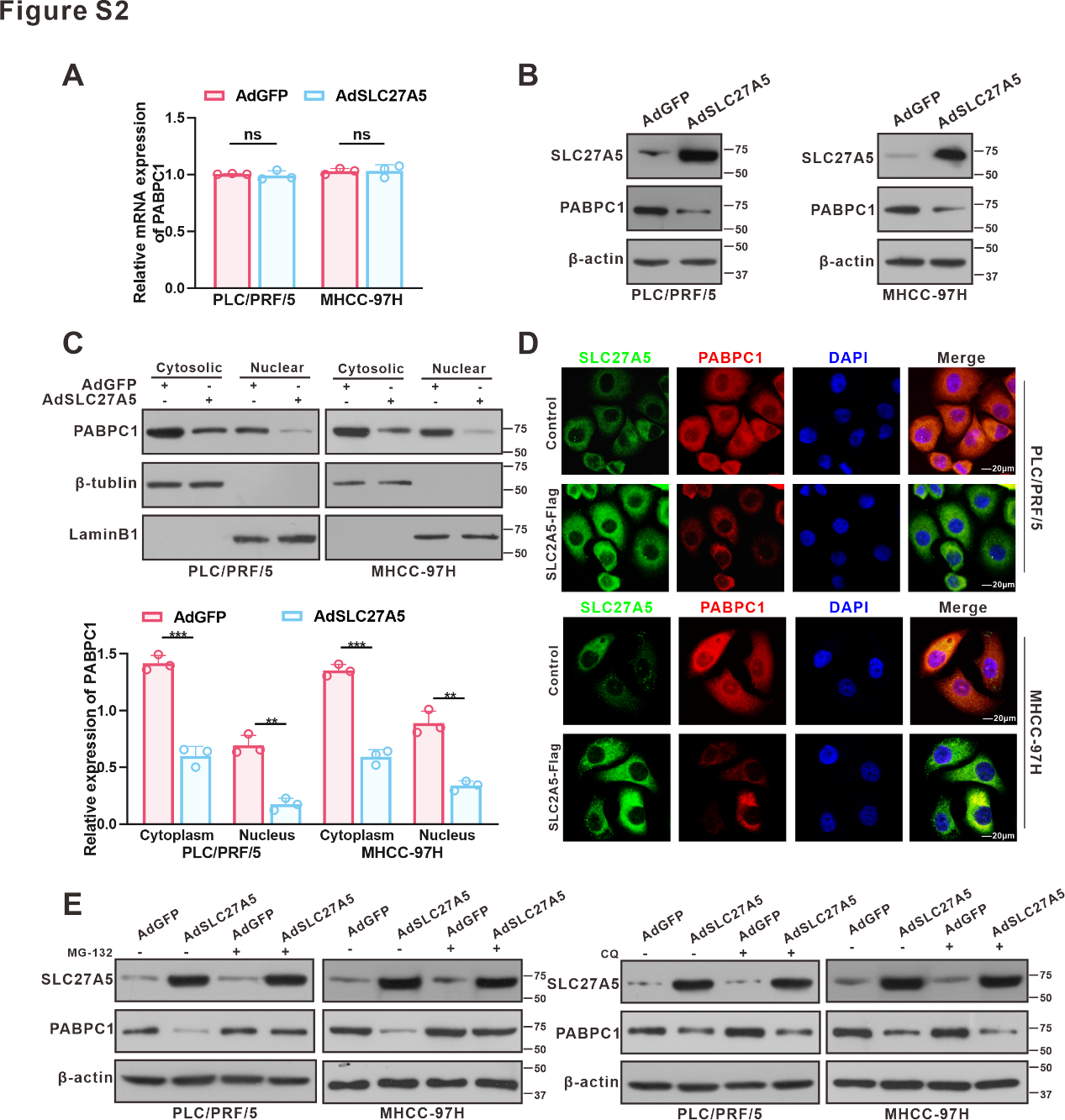


**Figure S2** SLC27A5 overexpression downregulates PABPC1 expression.

**(A)** RT-qPCR analysis of PABPC1 in PLC/PRF/5 and MHCC-97H cells infected with AdSLC27A5 (n = 3 independent replicates). **(B, C)** Immunoblots of changes in total cellular (B) and cytoplasmic and nuclear (C) PABPC1 protein levels in SLC27A5-OE cells. Data are shown as mean ± SEM. **p < 0.01, ***p < 0.001. **(D)** Immunofluorescence analysis of the cellular localization of PABPC1 in PLC/PRF/5 and MHCC-97H cells transfected with or SLC27A5-Flag (scale bar: 20 μm). **(E)** Immunoblots of SLC27A5 and PABPC1 protein levels in SLC27A5-OE cells treated with 10 μM MG132 or 50 μM chloroquine (CQ) before harvesting.


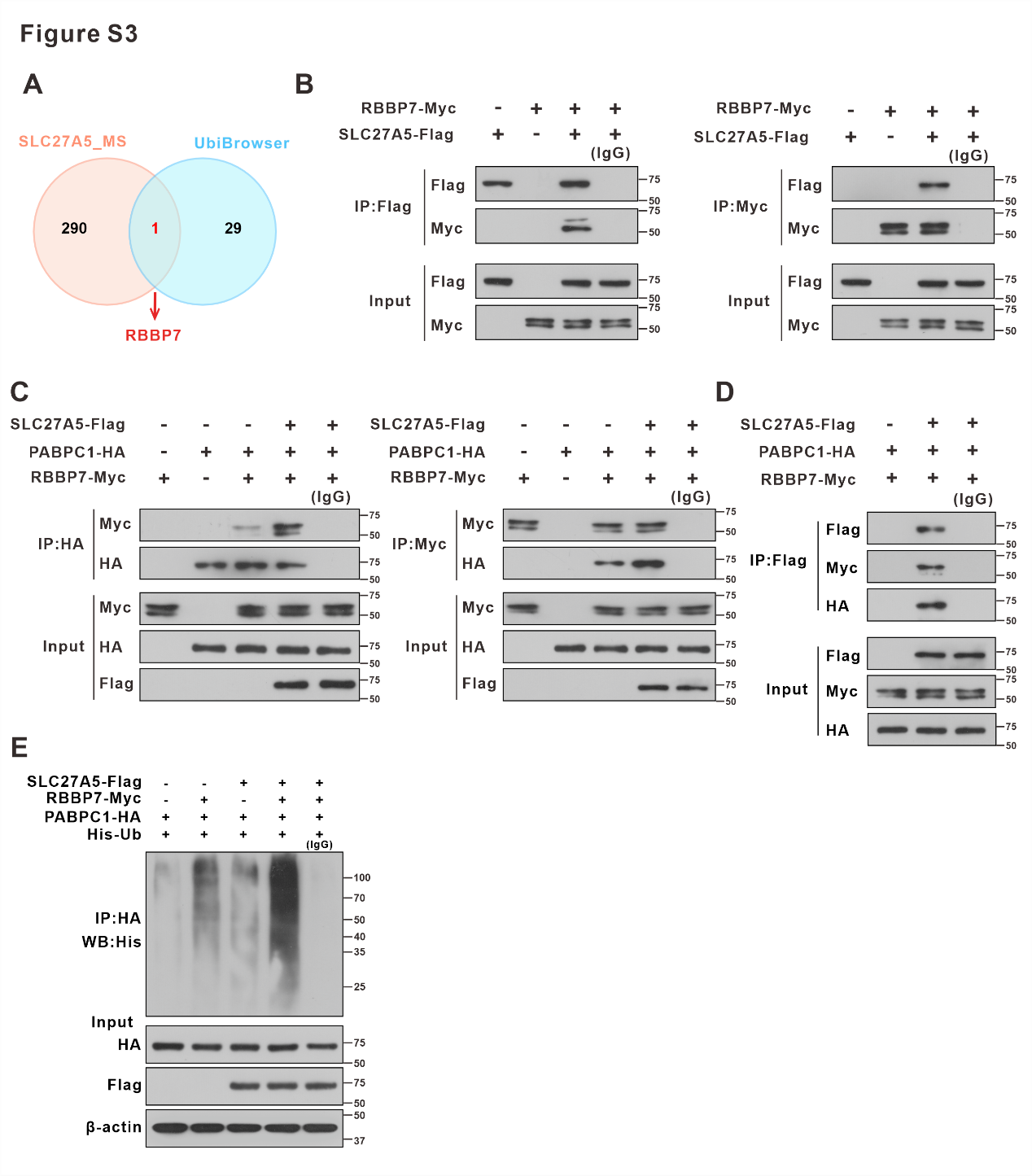


**Figure S3** SLC27A5 promotes the ubiquitination of PABPC1 via RBBP7. **(A)** UbiBrower database was used to predict the E3-ubiquitin ligase of PABPC1. Venn diagram of the UbiBrower database and the SLC27A5-interacting proteins. **(B)** Immunoblot analysis of Co-IP by anti-Flag (left) or anti-Myc (right) in HEK293 cells transfected with SLC27A5-Flag and RBBP7-Myc. **(C)** Co-IP of PABPC1-HA and RBBP7-Myc using an anti-HA antibody (left) or an anti-Myc antibody (right) in HEK-293 cells overexpressed SLC27A5. **(D)** Co-IP assays show the interaction among SLC27A5, PABPC1, and RBBP7 in HEK293 cells. Immunoprecipitations (IP) were performed with Flag-conjugated beads. Immunoblotting with anti-Flag, anti-Myc, or anti-HA was performed to analyze the immunoprecipitates. lgG served as a control. **(E)** In vivo PABPC1 ubiquitination assay was performed with HA-PABPC1, His-Ub, with or without Myc-RBBP7 transfection in SLC27A5-OE MHCC-97H cells. The reaction products were subjected to immunoblot analysis, and PABPC1 ubiquitination was detected with anti-His.

**
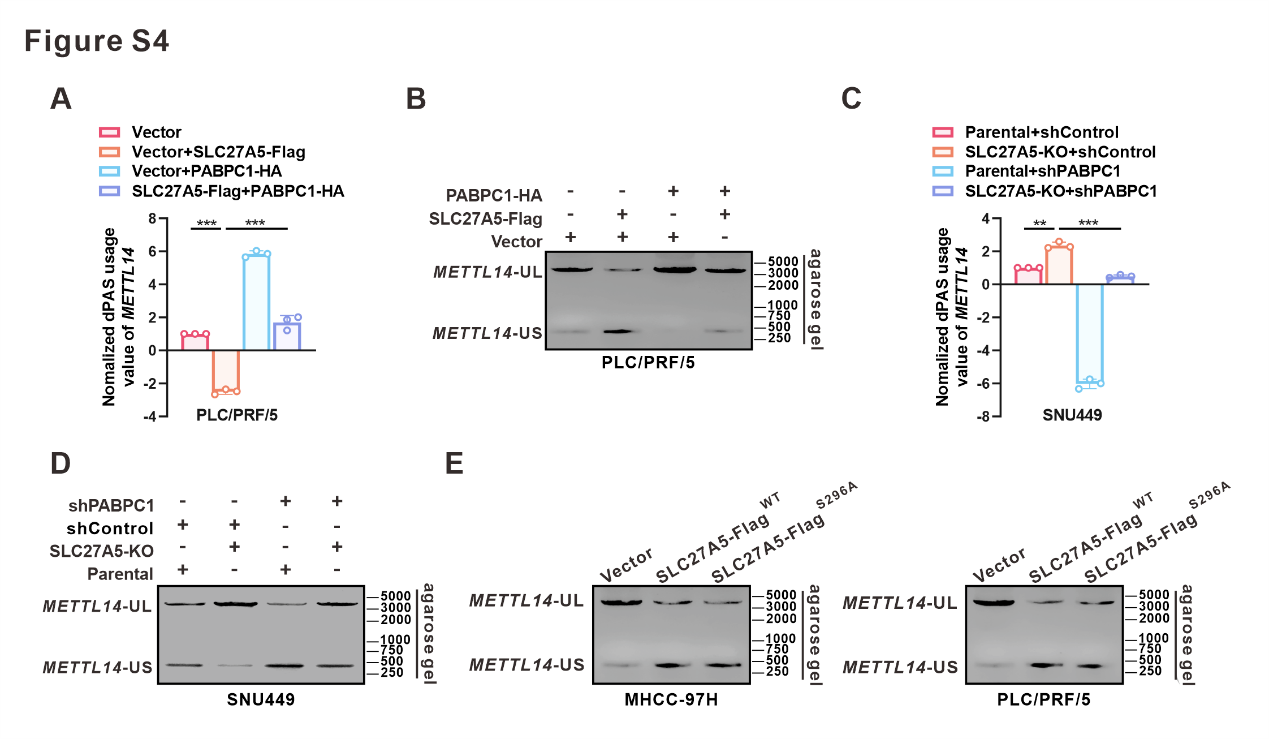
**

**Figure S4** SLC27A5 promotes *METTL14*-3'UTR shortening via PABPC1. **(A-D)** The qPCR and 3’RACE analysis showed changes in dPAS usage of *METTL14* and *METTL14*-UL/S expression in SLC27A5-OE PLC/PRF/5 cells transfected with PABPC1-HA or vector (A, B) and SLC27A5-KO SNU449 cells with PABPC1 knockdown (C, D). ***P* < 0.01; ****P* < 0.001. **(E)** 3’RACE analysis of *METTL14* in PLC/PRF/5 and MHCC-97H cells transfected with the recombinant plasmid SLC27A5-Flag (WT) or SLC27A5-Flag (S296A).

**
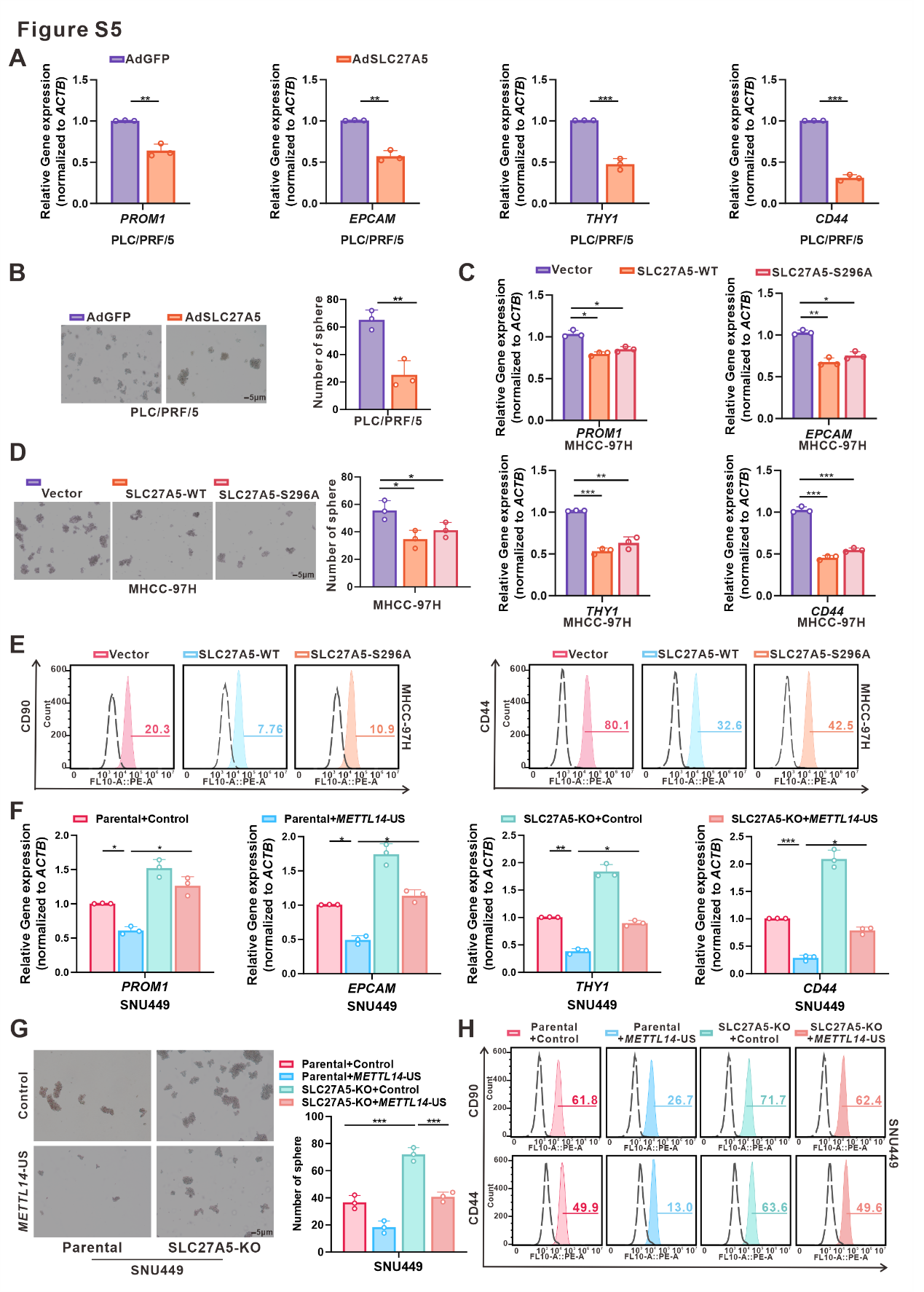
Figure S5** SLC27A5 inhibits HCC stemness via METTL14-US, not depending on its enzymatic activity. **(A, B)** SLC27A5-OE PLC/PRF/5 cells were used to detect the ability of stemness by qPCR analysis (A) and sphere-forming assays (B) (n = 3 technical replicates). **(C, D)** The ability of stemness was detected by qRT-PCR analysis (C) and sphere-forming assays (D) in MHCC-97H cells transfected with SLC27A5-Flag (WT) or SLC27A5-Flag (S296A). (n = 3 independent replicates). **(E)** Representative images of flow-cytometric analysis in the MHCC-97H cells transfected with SLC27A5-Flag (WT) or SLC27A5-Flag (S296A). **(F-G)** qRT-PCR analysis of stemness-related indicators (F) and sphere-forming assays (G) in SLC27A5-KO SNU449 cells transfected with a control vector or *METTL14*-US (n = 3 independent replicates). **(H)** Flow-cytometric analysis of the percentages of CD90^+^ or CD44^+^ cells in SLC27A5-KO SNU449 cells transfected with a control vector or *METTL14*-US. *P < 0.05, **P < 0.01, ***P < 0.001.


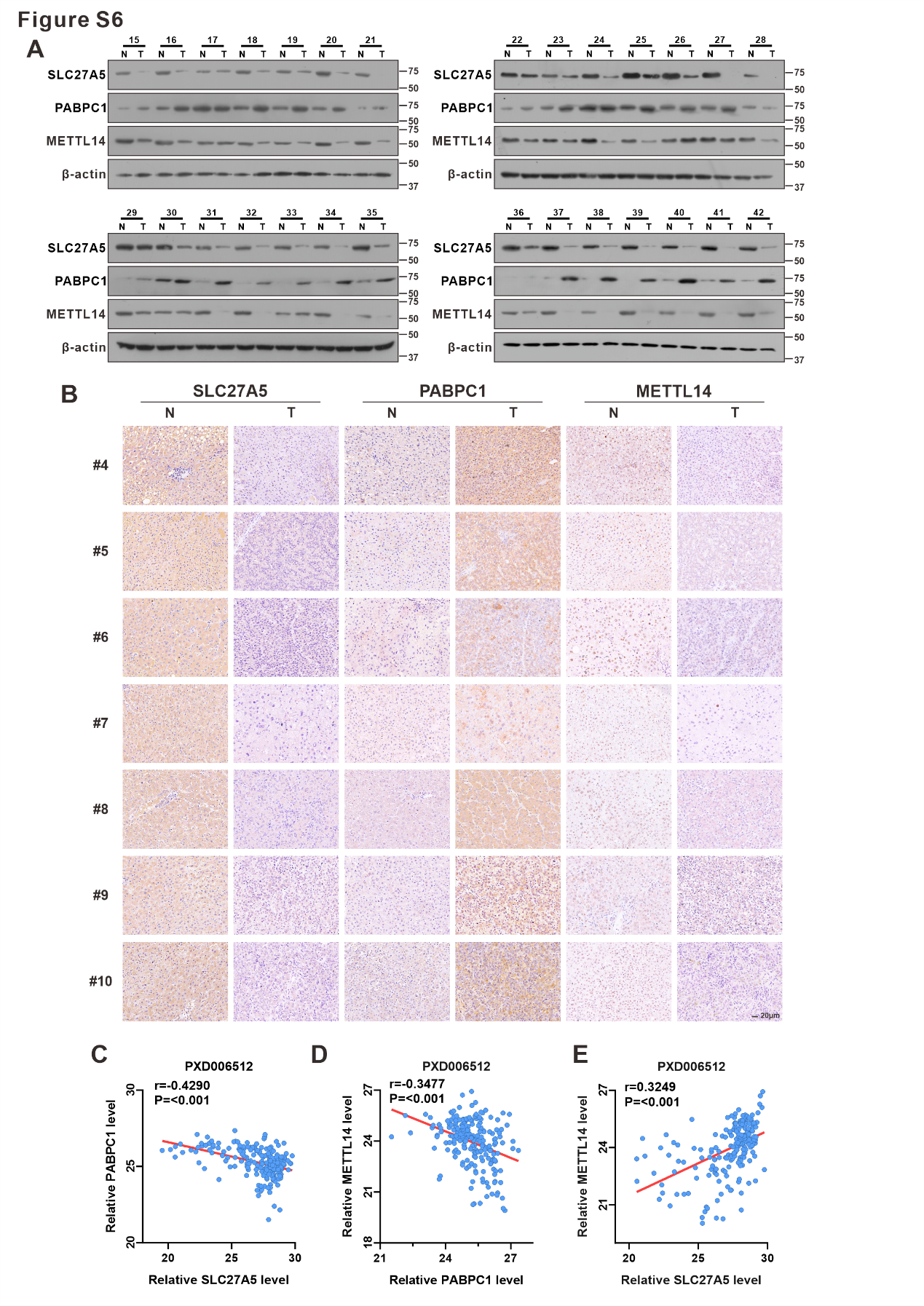


**Figure S6.** Correlations among SLC27A5, PABPC1, and METTL14 expression in HCC specimens. **(A)** Expression of SLC27A5, PABPC1, and METTL14 as detected by western blotting in representative human HCC and surrounding non-tumor tissues. **(B)** Expression of SLC27A5, PABPC1, and METTL14 in human liver tumor tissues and corresponding non-tumor tissues was detected by immunohistochemistry in seven HCC patients. **(C-E)** Correlation analysis of SLC27A5 and PABPC1 (C), PABPC1 and METTL14 (D), and SLC27A5 and METTL14 (E) protein levels based on the PXD006512 database (n = 222).

**Table S1** Clinical characteristics of HCC patients.

|  | **Sex** | **Age** | **HBsAg** | **HBeAg** | **HBcAb** | **AFP** | **HBV**  **DNA** | **ALT** | **AST** |
| --- | --- | --- | --- | --- | --- | --- | --- | --- | --- |
| 1 | female | 50 | positive | negative | positive | negative | positive | increase | increase |
| 2 | male | 59 | positive | negative | positive | negative | positive | increase | increase |
| 3 | male | 35 | positive | positive | positive | positive | positive | normal | increase |
| 4 | male | 59 | positive | positive | positive | positive | negative | normal | normal |
| 5 | male | 40 | positive | positive | positive | positive | positive | increase | increase |
| 6 | male | 36 | positive | positive | positive | positive | positive | increase | increase |
| 7 | male | 44 | positive | positive | positive | positive | negative | increase | increase |
| 8 | female | 55 | positive | positive | positive | positive | positive | normal | increase |
| 9 | male | 60 | positive | positive | positive | positive | positive | normal | normal |
| 10 | male | 52 | positive | positive | positive | positive | positive | normal | increase |
| 11 | male | 63 | positive | positive | positive | positive | positive | increase | increase |
| 12 | male | 38 | positive | positive | positive | positive | positive | increase | increase |
| 13 | male | 43 | positive | positive | positive | positive | positive | normal | increase |
| 14 | male | 71 | positive | negative | positive | negative | positive | increase | increase |
| 15 | male | 47 | positive | negative | negative | positive | positive | increase | increase |
| 16 | male | 65 | positive | positive | positive | positive | positive | increase | increase |
| 17 | male | 44 | negative | negative | positive | positive | positive | increase | increase |
| 18 | male | 37 | positive | negative | positive | negative | positive | increase | increase |
| 19 | male | 46 | positive | positive | positive | positive | positive | increase | increase |
| 20 | male | 57 | positive | positive | positive | positive | positive | increase | increase |
| 21 | female | 40 | negative | negative | negative | negative | - | increase | increase |
| 22 | male | 57 | positive | negative | negative | positive | positive | increase | increase |
| 23 | male | 55 | positive | positive | positive | positive | positive | increase | increase |
| 24 | male | 57 | positive | negative | positive | negative | - | increase | increase |
| 25 | male | 55 | positive | positive | positive | negative | positive | increase | increase |
| 26 | male | 59 | positive | positive | positive | negative | positive | normal | normal |
| 27 | male | 59 | positive | positive | positive | negative | positive | normal | normal |
| 28 | male | 55 | positive | positive | negative | negative | negative | normal | normal |
| 29 | male | 55 | positive | positive | positive | positive | positive | increase | increase |
| 30 | male | 48 | positive | negative | positive | positive | - | increase | increase |
| 31 | male | 63 | positive | negative | positive | negative | positive | increase | increase |
| 32 | male | 57 | positive | positive | positive | positive | positive | increase | increase |
| 33 | male | 62 | positive | negative | positive | positive | positive | increase | increase |
| 34 | female | 64 | negative | negative | positive | negative | - | increase | increase |
| 35 | male | 38 | positive | negative | positive | negative | positive | increase | increase |
| 36 | male | 50 | positive | positive | positive | positive | - | increase | increase |
| 37 | male | 59 | positive | negative | positive | negative | positive | increase | increase |
| 38 | male | 55 | positive | positive | positive | negative | negative | increase | increase |
| 39 | male | 57 | positive | positive | positive | positive | negative | increase | increase |
| 40 | male | 55 | positive | positive | positive | negative | positive | increase | increase |
| 41 | male | 47 | positive | negative | positive | negative | negative | increase | normal |
| 42 | male | 44 | positive | positive | positive | positive | - | increase | normal |

**Table S2** Primer sequences are used in this study.

| **Primers** | **Sequence(5’-3’)** | |
| --- | --- | --- |
| **RT-PCR** | **Forward (5’-3’)** | **Reverse (5’-3’)** |
| *SLC27A5* | TGATGGGACTTGTCGTTGGG | TATGTGTCCGGTCCTCTGGT |
| *β-actin* | AGGCCAACCGCGAGAAGATGACC | GAAGTCCAGGGCGACGTAGCAC |
| *METTL14-common* | TTTGATAGGGGAGGTGGAGAT | AGCGTGATTAGGAGTCATAGCC |
| *METTL14-distal* | TTTCCCACTGACCTTCCTCC | TCATCAACTACCTGCCCTCTTC |
| *EPCAM* | GTTCGGGCTTCTGCTTGC | GCCATTCATTTCTGCCTTCAT |
| *PROM1* | CTGATGCCTCTGGTGGGG | ACGCCTTGTCCTTGGTAGTGT |
| *THY1* | CTAGTGGACCAGAGCCTTCGT | GAGGACCTTCATGTTGTATTTGC |
| *CD44* | GGACTCTGCCTCGTGCCG | CGTGCCCTTCTATGAACCCA |
| **3'-RACE** | | |
| *METTL14-F1* | TTTGATAGGGGAGGTGGAGATTC | |
| *METTL14-F2* | GGGGTGGGTGAAAGAAGAGATAG | |
| **Fish mRNA probe** | | |
| *METTL14-3UL* | Cy3-CAACCGACAAAGCAGCCATA | |
| *hsa-miR-5009-3p* | FAM-TTTTGGACTTTCAGATTTAGGA | |
| **Plasmids** | **Forward (5’-3’)** | **Reverse (5’-3’)** |
| pSEB-3Flag-SLC27A5 | CGGAAGCTTATGGGTGTCAGGCAACAGTTGGCCTTG | CATGTCGACGAGCCTCCAGGTTCCCTCACA |
| pBu-3HA-PABPC1 | AGGGGTACCATGAACCCCAGTGCCCCC | GGAAGATCTCTAACAGTTGGAACACCGGTGG |
| pSEB-3Flag-SLC27A5 1-76aa | AGGGGTACCATGGGTGTCAGGCAACAGTTG | CCCAAGCTTCAGGAGGGTTAGTGCCAGGG |
| pSEB-3Flag-SLC27A5 77-690aa | AGGGGTACCATGGCACGGCTGCCCCCAGGA | CCCAAGCTTGAGCCTCCAGGTTCCCTCACA |
| pBu-3HA-PABPC1  1-370aa | AGGGGTACCATGAACCCCAGTGCCCCC | GGAAGATCTCTGCGCTGAGCTAAAGCTACATACA |
| pBu-3HA-PABPC1  371-636aa | AGGGGTACCATGAAAGAAGAGCGCCAGGCTC | GGAAGATCTCTAACAGTTGGAACACCGGTGG |
| pAdTrack-TO4-METTL14-CDS+US | TGGGGTACCATGGATAGCCGCTTGCAGG | ACGCGTCGACTGAGGGTTTTCCGTAGTTGGTT |
| pAdTrack-TO4-METTL14-CDS+UL | TGGGGTACCATGGATAGCCGCTTGCAGG | ACGCGTCGACTCACAAAGTGCTGGGATTAC |
| pGL3-Basic-METTL14-US | TGGGGTACCCCACCTCGATAATTGTTGAAGA | GGAAGATCTTGAGGGTTTTCCGTAGTTGGTT |
| pGL3-Basic-METTL14-UL | TGGGGTACCCCACCTCGATAATTGTTGAAGA | GGAAGATCTTCACAAAGTGCTGGGATTAC |
| pGL3-Basic-METTL14-UL-mutant | CATGATCTACCATTAAGCTAGAAAAGTTATTTAA | ATAACTTTTCTAGCTTAATGGTAGATCATGGCTC |
| ShPABPC1#1 | TGCATGAAGATGCACAGAAATTCAAGAGATTTCTGTGCATCTTCATGCTTTTTTC | TCGAGAAAAAAGCATGAAGATGCACAGAAATCTCTTGAATTTCTGTGCATCTTCATGCA |
| shPABPC1#2 | TGCTAGTCCTAGATTACTTATTCAAGAGATAAGTAATCTAGGACTAGCTTTTTTC | TCGAGAAAAAAGCTAGTCCTAGATTACTTATCTCTTGAATAAGTAATCTAGGACTAGCA |
| shIPABPC1#3 | TGCCTTAAGTGTGAAAGTAATTCAAGAGATTACTTTCACACTTAAGGCTTTTTTC | TCGAGAAAAAAGCCTTAAGTGTGAAAGTAATCTCTTGAATTACTTTCACACTTAAGGCA |

**Table S3** Antibodies and plasmids are used in this study.

| **Antibodies** | **Species** | **application** | **Concentration** | **Company source** | **Cat #** |
| --- | --- | --- | --- | --- | --- |
| SLC27A5 | Mouse | WB | 1:1000 | Gene Tex | GTX60688 |
| SLC27A5 | Mouse | IP | 1:100 | Novus Biologicals | NBP2-37412 |
| SLC27A5 | Mouse | IF | 1:500 | Gene Tex | GTX60688 |
| SLC27A5 | Mouse | IHC | 1:500 | Gene Tex | GTX60688 |
| PABPC1 | Rabbit | WB | 1:1000 | Proteintech | 10970-1-AP |
| PABPC1 | Rabbit | IP | 1:100 | Proteintech | 10970-1-AP |
| PABPC1 | Rabbit | IF | 1:50 | Proteintech | 10970-1-AP |
| PABPC1 | Rabbit | IHC | 1:200 | Proteintech | 10970-1-AP |
| METTL14 | Rabbit | WB | 1:1000 | Proteintech | 26158-1-AP |
| METTL14 | Rabbit | IF | 1:500 | Proteintech | 26158-1-AP |
| METTL14 | Rabbit | IHC | 1:500 | Proteintech | 26158-1-AP |
| HA-tag | Mouse | IP | 1:5000 | Invitrogen | 26183 |
| HA-tag | Mouse | IF | 1:10000 | Invitrogen | 26183 |
| Flag-tag | Mouse | IP | 1:5000 | Sigma-Aldrich | F3165 |
| Flag-tag | Mouse | IF | 1:10000 | Sigma-Aldrich | F3165 |
| GAPDH | Mouse | WB | 1:1000 | Beyotime | AF0006 |
| β-actin | Mouse | WB | 1:1000 | ZSGB-BIO | TA-09 |
| LaminB1 | Mouse | WB | 1:1000 | Proteintech | 66095-1-Ig |
| β-tublin | Mouse | WB | 1:1000 | Proteintech | 66240-1-Ig |
| Goat anti-rabbit IgG/TRITC, secondary | Goat | IF | 1:50 | ZSGB-BIO | ZF-0316 |
| Goat anti-mouse IgG/ TRITC, secondary | Goat | IF | 1:50 | ZSGB-BIO | ZF-0313 |
| Goat anti-rabbit IgG/FITC, secondary | Goat | IF | 1:50 | ZSGB-BIO | ZF-0311 |
| Goat anti-mouse IgG/FITC, secondary | Goat | IF | 1:50 | ZSGB-BIO | ZF-0312 |
| PE anti-mouse/human CD44 | Rat | FC | 1:100 | Biolegend | 103007 |
| PE anti-mouse/human CD90 | Rat | FC | 1:100 | Biolegend | 328109 |
| **Plasmids** | | **Company source** | | **Cat #** | |
| pLV2-CMV-RBBP7(human)-Myc | | MiaoLingBio | | P44733 | |
